# The Type II Secreted Lipase/Esterase LesA is a Key Virulence Factor Required for *Xylella fastidiosa* Pathogenesis in Grapevines

**DOI:** 10.1038/srep18598

**Published:** 2016-01-12

**Authors:** Rafael Nascimento, Hossein Gouran, Sandeep Chakraborty, Hyrum W. Gillespie, Hebréia O. Almeida-Souza, Aye Tu, Basuthkar J. Rao, Paul A. Feldstein, George Bruening, Luiz R. Goulart, Abhaya M. Dandekar

**Affiliations:** 1 Plant Sciences Department, University of California, Davis, 1 Shields Ave, Davis CA, 95616, USA.; 2 Plant Pathology Department, University of California, Davis, 1 Shields Ave, Davis CA, 95616, USA.; 3 Medical Microbiology and Immunology Department, University of California, Davis, 1 Shields Ave, Davis CA, 95616, USA; 4 Department of Biological Sciences, Tata Institute of Fundamental Research, Homi Bhabha Road, Mumbai 400 005, India; 5 Institute of Genetics and Biochemistry, Federal University of Uberlândia, Av. Amazonas, Bloco 2E, Campus Umuarama, 38400-902, Uberlândia MG, Brazil

## Abstract

Pierce’s disease (PD) of grapevines is caused by *Xylella fastidiosa* (*Xf*), a xylem-limited gamma-proteobacterium that is responsible for several economically important crop diseases. The occlusion of xylem elements and interference with water transport by *Xf* and its associated biofilm have been posited as the main cause of PD symptom development; however, *Xf* virulence mechanisms have not been described. Analysis of the *Xf* secretome revealed a putative lipase/esterase (LesA) that was abundantly secreted in bacterial culture supernatant and was characterized as a protein ortholog of the cell wall-degrading enzyme LipA of *Xanthomonas* strains. LesA was secreted by *Xf* and associated with a biofilm filamentous network. Additional proteomic analysis revealed its abundant presence in outer membrane vesicles (OMVs). Accumulation of LesA in leaf regions associated positively with PD symptoms and inversely with bacterial titer. The lipase/esterase also elicited a hypersensitive response in grapevine. *Xf lesA* mutants were significantly deficient for virulence when mechanically inoculated into grapevines. We propose that *Xf* pathogenesis is caused by LesA secretion mediated by OMV cargos and that its release and accumulation in leaf margins leads to early stages of observed PD symptoms.

*Xylella fastidiosa* (*Xf*) is a fastidious, xylem-limited gamma-proteobacterium that causes several economically important diseases in crop plants, including grapevine, citrus, periwinkle, almond, oleander, and coffee^1^,^2^. In the field, *Xf* is vector-transmitted by various xylem sap-feeding sharpshooter insects^3^,^4^. The *Xf* subspecies *fastidiosa* (*Xff*), exemplified by the California strain Temecula1, causes Pierce’s disease (PD) in grapevine. PD poses a great threat to the winegrowing regions of California^5^. However, the *Xf* life cycle and virulence mechanism are not entirely understood^2^. A characteristic PD symptom is marginal leaf chlorosis progressing to necrosis (leaf scorch). Three general explanations for PD symptom development have been proposed: (i) occlusion of xylem elements and interference with water transport by *Xf* and its associated biofilm, (ii) plant systematic responses, e.g., growth regulator imbalance, and (iii) *Xf*-generated phytotoxin^5^. The occlusion hypothesis extends from early observations of xylem element blockage in PD^6^. Generally, plant species and regions of the plant body that show the most severe symptoms are those with the greatest proportion of colonized vessels^3^,^7^,^8^,^9^,^10^,^11^, in agreement with the occlusion hypothesis. PD and other *Xf* diseases are associated with decreased leaf water potential^12^ and altered carbon isotope incorporation^13^, also consistent with water stress. Although the occlusion hypothesis widely considered to be supported, other observations are inconsistent with this explanation. The symptoms of wilting and PD are not similar and are additive in effect, *Xf* accumulation in grapevine leaves does not correlate with local PD symptom severity, and in some infections of grapevine and other *Xf* hosts, severe necrotic symptoms are associated with only minimal vessel occlusion^5^.

The secretion of virulence factors by pathogens is an important trigger mechanism for many plant diseases. Unlike closely related pathogens from the genus *Xanthomonas*, *Xff* lacks the type III secretion system (T3SS)^14^. However, *Xanthomonas* and *Xf* have in common a similar type II secretion system (T2SS) for a battery of important extracellular enzymes involved in nutrient acquisition and virulence^15^. In *Xff*, genes have been identified that code for plant cell wall degrading enzymes (CWDEs) such as polygalacturonase, cellulase, and proteases^14^,^16^,^17^. These enzymes may aid *Xff* migration inside xylem vessels by degrading the pit membrane and releasing the carbohydrates necessary for bacterial growth and survival^17^. Cell wall degradation by CWDEs releases oligosaccharides as a bacterial nutrient source, which can also induce potent plant innate immune responses leading to cell death. Such plant defense responses include production of phytoalexins, fortification of cell walls through callose deposition, oxidative burst, and induction of programmed cell death^18^,^19^,^20^.

We report here our analysis of the *Xff* Temecula1 secretome, including comparison with the bacterial surfaceome and outer membrane and outer membrane vesicle proteomes. The uncharacterized protein PD1703 was identified as the most abundant secreted protein and its characteristics and likely participation in *Xff*-initiated symptoms of PD are discussed.

## Results

### Lipases are a highly abundant component of the *Xff* secretome

**Figure 1:**
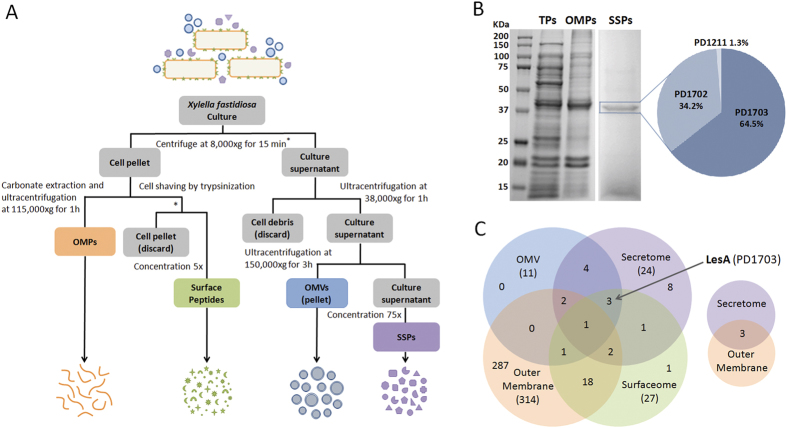
*X. fastidiosa* subcellular proteomic analysis. (**A**) Workflow describing the procedures used to isolate *X. fastidiosa* Temecula1 outer membrane proteins (OMPs), outer membrane vesicles (OMVs), surface peptides (surfaceome) and soluble supernatant proteins (SSPs). Outer membrane proteins were extracted using 0.1 M sodium carbonate buffer (pH 11.0) followed by mass spectrometry. To isolate surface peptides, cells (4 × 10^8^ cells/mL) were harvested from a four- to six- day-old culture and subjected to tryptic digestion (cell shaving). Peptides were concentrated (5×) and subjected directly to LC/MSMS. OMVs were purified from the culture supernatant using two ultracentrifugation steps (38,000 × *g* for 1 h to pellet cell debris followed by 150,000 × *g* for 3 h for OMVs precipitation). The remaining supernatant was concentrated (∼75 to 100×) using Amicon Ultra-15 3 K filter units to identify SSPs by mass spectrometry. (**B**) SDS-PAGE 12% resolution of 10 μg *Xff* total protein (TP), OMP (Table S2) and SSPs (Table 1). Three putative lipase/esterases (PD1703, PD1702, and PD1211) were identified in the highlighted band, which was composed mostly of PD1703 (LesA) and PD1702. (**C**) Venn diagram quantifying the number of proteins identified in each subcellular proteome. The major outer membrane protein MopB was found in all samples, although it was very poorly represented in the secretome. The lipase/esterase LesA was found in the secretome, surfaceome and OMV proteome.

The bacterial proteome was investigated to characterize the subcellular localization of *Xff* proteins as described (Fig. 1A). The identities of soluble supernatant proteins (SSPs) in culture medium were determined after removing bacterial cells and cell fractions through a series of ultracentrifugation and concentration steps. Twenty-four SSPs were identified by LC-MS/MS in the culture supernatant (Table 1). These include many potential plant cell wall-degrading enzymes necessary for nutrient acquisition such as lipases/esterases (PD1703, PD1702 and PD1211), proteases (PD0313, PD0657, PD0950, PD0956 and PD1850) and cellulase (PD0529). SSPs potentially related to cell adhesion (PD1792 and PD0731), bacterial toxicity (PD1427), and pathogenesis (PD1506 and PD0855) were also identified. Separation of SSPs proteins by one-dimensional SDS-PAGE (Fig. 1B) revealed a prominent 40 to 50 KDa band, indicating that a few proteins were highly abundant in *Xff* secretome. After in-gel digestion, mass spectrometry analysis of this particular band revealed two 42 KDa (PD1703, PD1702) proteins and one 46 KDa (PD1211) putative uncharacterized protein. The high amino acid sequence homology of these three proteins allowed us to estimate their relative abundance from their numbers of assigned spectra (Fig. S1). PD1703 (64.5%; 783) was more abundant in the secretome than PD1702 (34.2%; 268) and PD1211 (1.3%; 10) (Fig. 1B). *In silico* analysis predicted that PD1703 is a secreted lipase/esterase protein with high sequence similarity to the cell wall-degrading enzyme LipA from *Xanthomonas oryzae* pv. *oryzae* (*Xoo*)^21^ and *Xanthomonas campestris* pv. *vesicatoria* (*Xcv*)^22^. To confirm the lipase/esterase functions of PD1703, we evaluated the enzymatic activity of *Xff* SSPs against short- and long-chain substrates. As reported for *Xoo* LipA^21^, *Xff* SSPs degraded *p*-nitrophenyl butyrate and the short-chain triacylglyceride tributyrin (C4), but not the long-chain triacylglyceride tricaprin (C10) (Fig. S2).

### *Xff* secretes LesA as cargo in outer membrane vesicles

**Figure 2:**
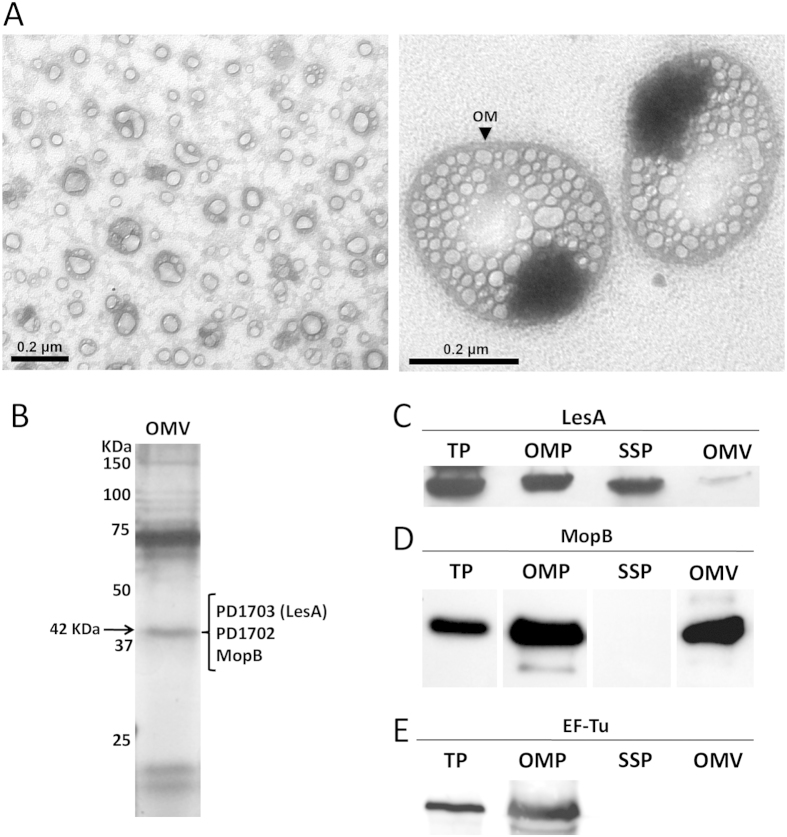
*X. fastidiosa* outer membrane vesicle (OMV) visualization and protein cargo analysis. (**A**) Electron micrograph of negatively stained outer membrane vesicles extracted from the *Xff* Temecula1 cell free supernatant. Both small (left; <200 nm diameter) and large (right; up to 400 nm diameter) OMVs were identified. The black arrow indicates the outer membrane, which was released from the bacterial cell, presumably enclosing periplasmic content to produce the OMV. Large OMVs contained an electrodense material of unknown composition that was not seen in small OMVs. (**B**) Silver-stained SDS-PAGE 12% separation of OMV cargo showing a band at apparent molecular weight 42 KDa, presumed to be composed of proteins LesA, PD1702, and MopB, all found in the OMV proteomic analysis (Table S1). (**C–E**) Immunoblot detection of LesA, MopB, and EF-Tu in *Xff* total proteins (TP), outer membrane proteins (OMP), soluble supernatant proteins (SSP), and outer membrane vesicle proteins (OMV). For TP, OMP and SSP (defined in Fig. 1A), 10 μg protein was loaded in the gel; however, only 2 μg protein was loaded in OMV due to the high non-protein content of this sample. This may explain the weak detection of LesA in C.

Gram-negative bacteria produce outer membrane vesicles (OMVs) that contain biologically active proteins and perform diverse biological functions^23^,^24^. Production of OMVs by *Xff* was recently reported elsewhere^25^. The complete OMV protein cargo has been described for many Gram-negative bacterial species^23^, including *Xff*’s closely related pathogen *Xanthomonas campestris* pv. *campestris* (*Xcc*)^26^, but not for *Xff*. The OMV fraction was negatively stained and examined using transmission electron microscopy (TEM) (Fig. 2A), revealing the expected vesicles. Our investigation of OMV protein cargo by LC-MS/MS identified 11 proteins (Table 2). Interestingly, the most abundant SSP, LesA (PD1703), was also part of the OMV cargo. Immunoblot analysis confirmed the localization of *Xff* LesA in the secretome and OMV proteome (Fig. 2C). In addition to LesA, the secreted proteins PD1702, PD0731 and PD1427 were identified in the OMV cargo, indicating that delivery of these proteins in the host could also be mediated by OMVs. Four outer membrane proteins (PD1709, PD1807, PD0313 and PD1283) were identified in the *Xff* OMV proteome. We previously identified MopB (PD1709) as the major *Xff* outer membrane protein (OMP)^27^. PD1807 and PD1283 were also found in our OM preparation (Table S2). The presence of MopB in both the *Xff* OM and OMV was confirmed by immunoblot (Fig. 2D). The elongation factor Tu (EF-Tu), which is a PAMP in many gram-negative bacteria^28^,^29^, was abundant in the *Xff* outer membrane fraction, but was not identified in *Xff* secretome or as part of the OMV cargo (Table S2; Fig. 2E).

### LesA is localized in the secreted filamentous network

**Figure 3:**
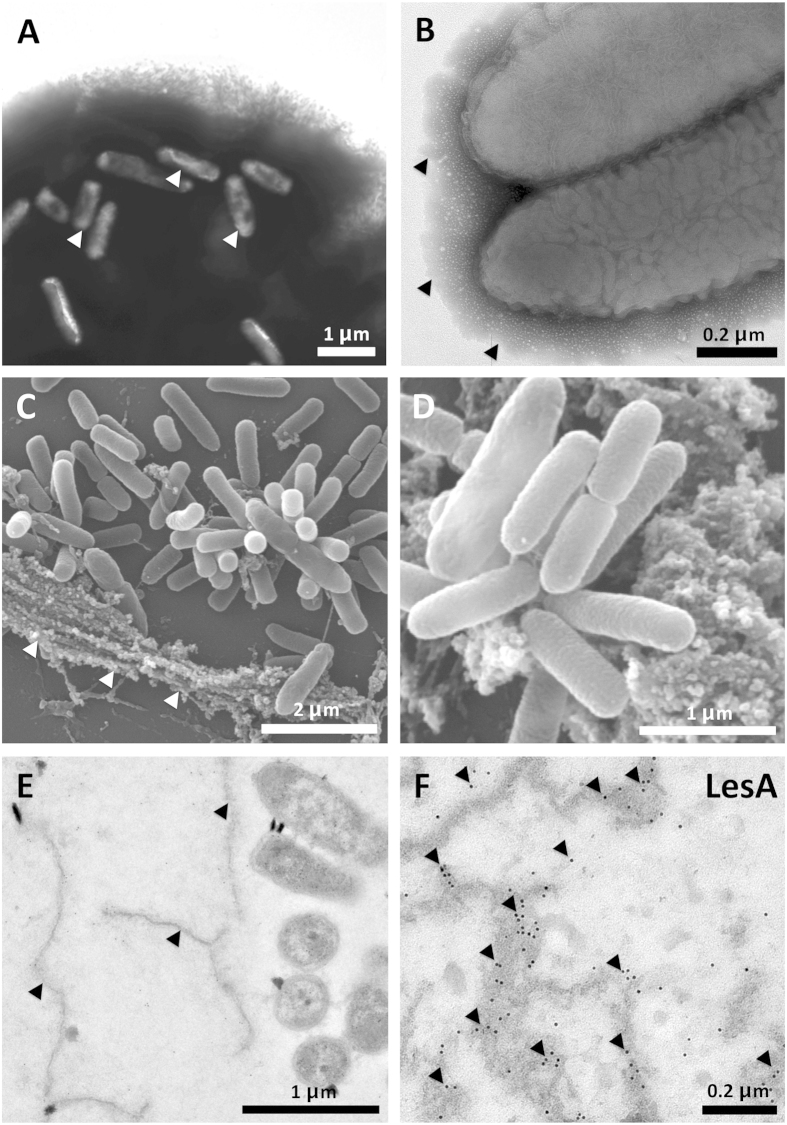
Electron microscopy of *Xff* cells and the secreted filamentous network. (**A**) Negative staining of *Xff* aggregates showing abundant secreted material in which bacterial cells (white arrow) are embedded. (**B**) Closer view of the secreted material surrounding planktonic *Xff* cells isolated from the same culture shown in A. (**C,D**) Scanning electron microscopy of *Xff* showing bacterial cells surrounded by the secreted filamentous network (white arrows). (**E,F**) Immunogold detection of LesA in the secreted filamentous network (black arrows) surrounding *Xff* cells (E). LesA was abundant in the secreted network as shown in F (black arrows).

To visualize the distribution pattern of *Xff*-secreted material, we examined bacterial cells in culture using scanning and transmission electron microscopy (SEM and TEM). Negatively stained cell clusters show that *Xff* produces cell aggregates embedded in a dense secreted material possibly composed of extracellular polysaccharide (EPS) and proteins that are weakly attached to the bacterial surface (Fig. 3A). The same dense material surrounds planktonic cells (Fig. 3B). SEM analysis of the bacterial culture revealed that *Xff* secretes a filamentous network of unknown composition similar to the matrix seen surrounding cells aggregates in the xylem vessels of infected grapevines^30^ (Fig. 3C,D). TEM analysis of the secreted matrix revealed its close localization to *Xff* cells *in vitro* (Fig. 3E). Immunogold labeling and TEM revealed that LesA is embedded in the *Xff* secreted network (Fig. 3F).

### LesA accumulates abundantly in leaf regions with minimal *Xff* titer and is associated with early stages of PD symptom development

Although LesA accumulated in both OMVs and the secreted matrix of *Xff* cells, its localization in *Xff*-infected host tissue was unknown. To search for LesA and other potential secreted virulence factors in the infected host, we compared the total leaf proteome of *Xff*-infected and non-infected grapevines. Of the 524 proteins found, six were of *Xff* origin (Table 3). LesA (the most abundant secreted protein) and MopB (the most abundant outer membrane protein) were identified in infected host tissue. The surface protein Hsf (PD0744) was found in infected grapevine leaves, although it was not previously identified in the secretome of cultured *Xff*. This suggests that both expression and secretion of Hsf may be triggered during infection of grapevine leaves. As expected, no *Xff* protein was found in non-infected grapevine leaves.

**Figure 4:**
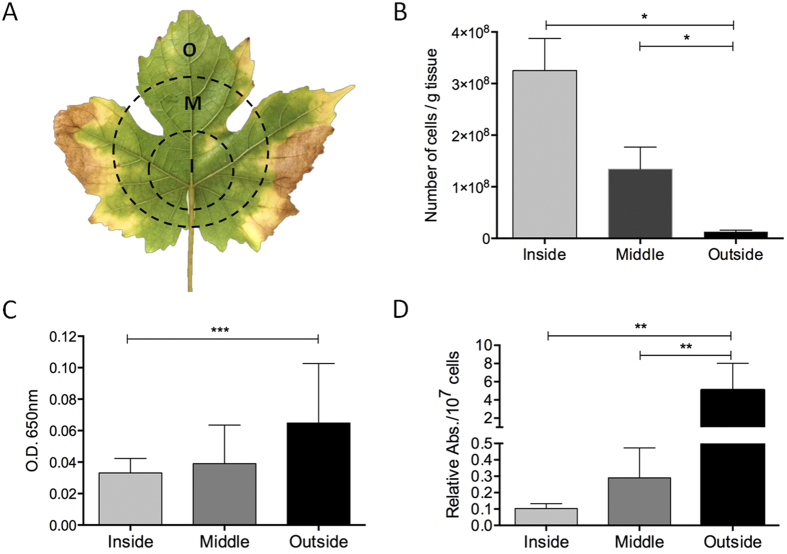
Detection of LesA movement in grapevine leaves. (**A**) Grapevine leaf showing the three segments, each 1 to 2 cm in the radial direction, used to detect LesA: I (inside), M (middle) and O (outside). (**B**) Viable *Xff* cell assay of leaf segment (n = 3) extracts revealed ~10-fold reduction in the bacterial population at the leaf edges (outside) than at the center (inside). The bacterial titration was carried out using a standard curve of five dilutions (Fig. S4; *One-way ANOVA test; R^2^ = 0.9817; p < 0.01). (**C,D**) The amount of LesA detected in grapevine leaves differs slightly in the inside and outside segments (C) (***Mann-Whitney test; p = 0.1). However, the relative abundance of LesA per number of *Xff* cells (D) is significantly greater in the outside area than in middle and inside segments (**One-way ANOVA test; p < 0.05), suggesting that the secreted LesA is moving from the *Xff*-crowded inside area to the symptomatic leaf extremities where the bacteria is scarcely present. This experiment was conducted twice with similar results. Picture of leaf was taken by Rafael Nascimento.

The accumulation of LesA in *Xff*-infected host tissue confirmed our hypothesis that LesA is highly expressed and secreted not only *in vitro* but also during the *Xff* infection/colonization process. We postulated that LesA has an important role in *Xff* pathogenesis in grapevines similar to that seen in *Xoo*, where it elicits callose deposition and programmed cell death in rice^21^,^31^. We evaluated whether the presence and abundance of LesA correlates with PD symptom development in grapevine leaves 12 weeks post inoculation (wpi). We divided infected leaves into three major areas (inside, middle and outside) corresponding to the pattern of symptom development (Fig. 4A). Viable *Xff* cells were abundant in the inside portion of infected leaves, but were less abundant in the middle and outside areas, especially close to the leaf margins (Fig. 4B). The most characteristic early PD symptom in grapevines, the marginal leaf necrosis called leaf scorch, was associated with low *Xff* titer as previously reported^32^. We postulate that *Xff* could be releasing either LesA-containing OMVs or SSP, or both, from its colonized region near the petiole. These could deliver LesA to the leaf margins, causing PD symptoms. To test this hypothesis, we localized LesA using a polyclonal antibody in the three areas. LesA was slightly more abundant in the leaf edges than the middle and inside portions (Fig. 4C). When the amount of LesA per viable *Xff* cell was calculated, a difference of ∼100-fold (p < 0.05) was observed (Fig. 4D).

### LesA elicits a hypersensitive response in grapevine

**Figure 5:**
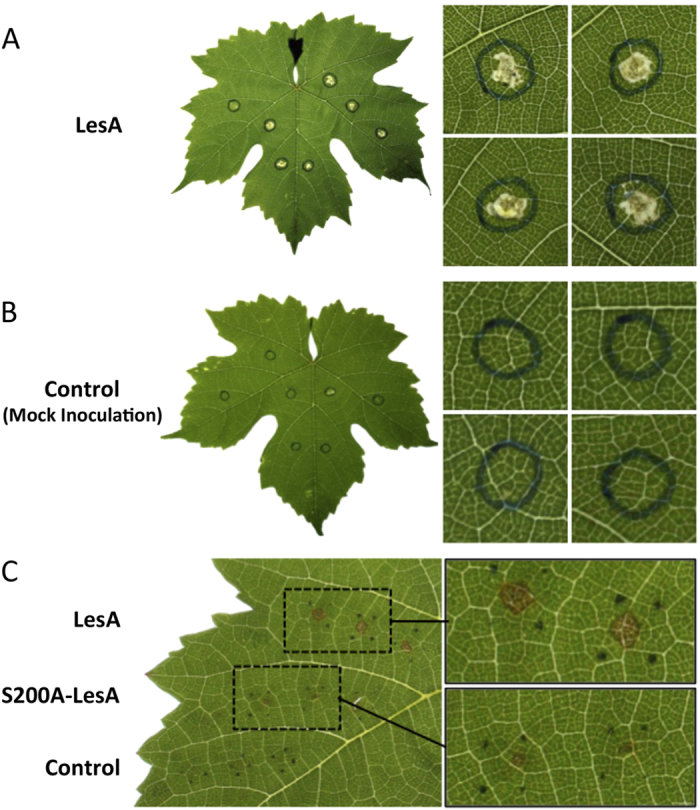
LesA elicits HR-like symptoms in grapevine leaves. Crude protein from LesA-expressing *E. coli* was isolated and syringe-infiltrated in grapevine leaves of greenhouse-grown plants. (**A**) HR-like symptoms appeared 24 h after infiltration. (**B**) No reaction was observed after infiltration of crude protein from *E. coli* expressing the empty vector (pJexpress 401). (**C**) Reduced HR-like symptoms caused by S200A-LesA, in which the residue Ser-200 from *Xff* LesA catalytic triad was replaced by alanine. Wild-type LesA caused the same necrosis shown in A. Pictures of leaves were taken by Hossein Gouran.

LipA from *Xoo* elicits an innate immune response in rice mediated by cell wall degradation, induces callose deposition, and triggers programmed cell death^21^,^31^. In addition, LipA is required for wild-type levels of *Xcv* virulence on tomato^22^. Analysis of the high-resolution crystal structure of *Xoo* LipA revealed the canonical catalytic triad residues Ser-176, Asp-336 and His-377. Ser-176 is embedded in the hydrolase conserved motif Gly-X-Ser-X-Gly. Moreover, mutation in the residue Ser-176 reduced *Xoo* virulence on rice, confirming that the enzymatic activity of LipA, rather than LipA as a protein, is essential for optimal virulence^21^. *In silico* analyses mapped LesA from Temecula1 (PD1703) and 9a5c (XF0357) *Xf* strains on the *Xoo* LipA structure, assigning the catalytic triad of both *Xf* LesA molecules to residues Ser-200, Asp-360 and His-402 (Fig. S1). To test whether LesA contributes to *Xff* virulence in grapevine, we expressed both *Xff* LesA and its alanine substitution mutant S200A-LesA in *E. coli* and pressure-infiltrated leaves with protein extract from *E. coli*. Heterologous expression of both LesA and S200A-LesA constructs was confirmed by immunoblot detection. Extracts from LesA-producing *E. coli*, but not from the empty-vector control, induced necrosis in the infiltrated area, indicating that LesA may contribute to *Xff* pathogenesis in grapevines (Fig. 5A,B). Interestingly, infiltrated *E. coli* extracts producing the *Xff* S200A-LesA mutant elicited only limited HR-like symptoms compared to wild-type LesA (Fig. 5C), consistent with enzymatic activity of *Xff* LesA being essential for maximal virulence, as for *Xoo* LipA.

### LesA is required for virulence of *X. fastidiosa* in grapevines

**Figure 6:**
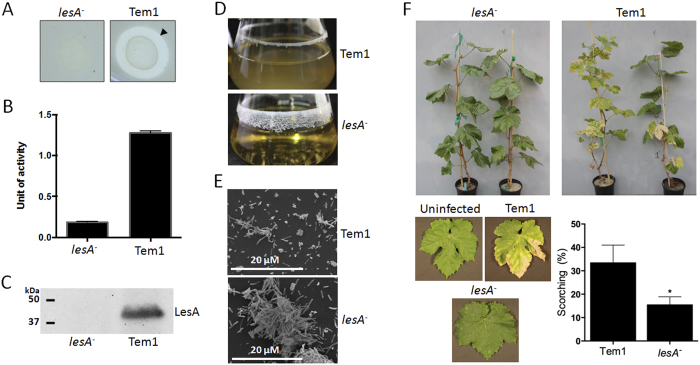
*X. fastidiosa lesA* mutants are deficient in virulence. (**A**) Lipase activity was analyzed around *Xff lesA* mutant (*lesA*^-^) and WT (Tem1) colonies grown in PD3-agar medium supplemented with 1% trybutirin. (**B**) Esterase activity of proteins from *lesA*^*-*^ and WT by 4-methylumbelliferyl butyrate (4-MUB) assay. Esterase activity is diminished significantly in the *lesA* mutant. (**C**) Immunoblot detection of LesA in *Xff lesA*^*−*^ and WT total proteins. The LesA protein was not detected in the mutant, confirming the mutagenesis of the *lesA* gene. (**D**) Analysis of biofilm formation in liquid culture, showing the predominance of the biofilm growth mode in *lesA*^*−*^ when compared to WT. (**E**) Scanning electron micrographs of *Xff* culture showing the aggregation of *lesA*^*−*^ cells typically found in biofilms. (**F**) Pierce’s disease symptoms in grapevines. Thompson Seedless infected with the wild-type strain Temecula1, the *lesA*^*−*^ strain, and uninfected (mock inoculated). Data is shown as the difference from uninfected controls (average of 26 plants). Plants inoculated with *Xff lesA*^*−*^ strain have significantly less PD symptoms (*One-Tailed T-test with Welch’s correction and p value of 0.0351) than Temecula1 infected plants. Pictures were taken by Hossein Gouran.

To test whether LesA has a role in *Xff* virulence, we generated a *lesA* mutant using homologous recombination and compared the performance of both proteins after inoculation in grapevines. Our *Xff lesA* mutant lacked lipase and esterase activities (Fig. 6A,B) and expresses no LesA protein (Fig. 6C). Interestingly, the *Xff lesA* mutant was predominantly found in the biofilm mode of growth when cultivated in liquid media, unlike the wild-type strain (Fig. 6D), and formed large cell aggregates (Fig. 6E). The inability of the *lesA* mutant to elicit typical PD symptoms was confirmed by significantly reduced symptomatic leaves (50%; p < 0.05) observed in infected grapevines compared with plants inoculated with the parental strain (Fig. 6F).

### Wild-type *Xff* and its quorum-sensing mutants have distinct patterns of expression for *lesA*

**Figure 7:**
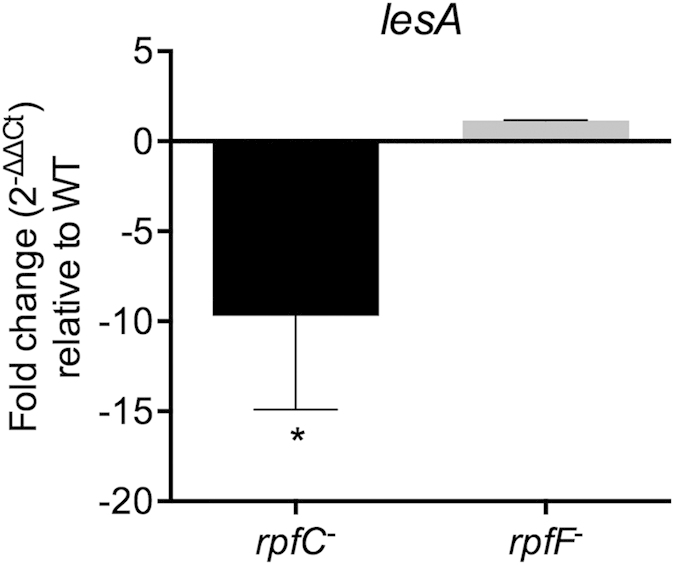
LesA is down regulated in an *Xff* quorum-sensing mutant. Comparison of RNA accumulation in mutants and wild-type by RT-PCR revealed that *lesA* is regulated by *rpfC*. RNA was extracted from *Xff* cells grown in PD3 medium (three flasks/condition; 10^7^–10^8^ cells/mL) at 28 °C and 120 rpm. The 16S rRNA gene was used as an endogenous control. Unpaired t test with Welch’s correction was used for statistical analysis (one-tailed; *p < 0.01).

Cell–cell signaling plays an important role in the virulence of many plant pathogenic bacteria. *Xff* produces the quorum-sensing signaling molecule DSF (diffusible signaling factor) that regulates bacterial pathogenesis. In *Xanthomonas campestris* pv. *campestris* (*Xcc*) and *Xanthomonas oryzae* pv. *oryzae* (*Xoo*), DSF-deficient mutants have reduced virulence^33^,^34^. *Xff rpfF* mutants deficient in DSF production possess a hypervirulent phenotype when inoculated into grapevines^30^. The *rpfF* gene is required for DSF production in both *Xff* and *Xanthomonas* species. Interestingly, *Xff rpfC* mutants overproduce DSF and have a hyperattachment phenotype, which makes them deficient in virulence and movement in grapevine xylem vessels^35^. For specific virulence-related genes, messenger RNA accumulation for *rpfC* and *rpfF* mutants and the wild type was assessed by RT-PCR. The *lesA* gene was down regulated in the *rpfC* mutant (Fig. 7).

### *Agrobacterium* harboring *X. fastidiosa* LesA is hypervirulent

**Figure 8:**
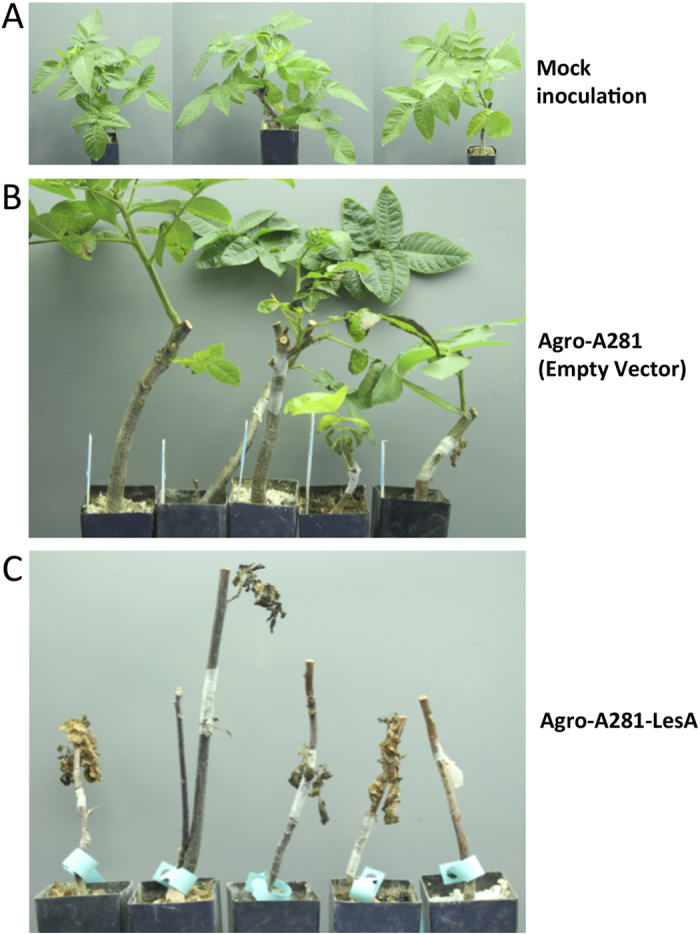
Virulence assay in walnut plants. *Xff lesA* gene was expressed in *A. tumefaciens* under the control of its own promoter. *Agrobacterium* harboring *Xff* LesA became hypervirulent. Walnut plants inoculated with agrobacterium expressing *Xff* LesA (Agro-A281-LesA), but not from the mock inoculation (PBS) and empty vector (**A,B**), presented the first signs of disease at eight wpi and were dead at 12 wpi (**C**). Pictures were taken by Hossein Gouran.

To assess whether LesA increases *A. tumefaciens* virulence, we expressed the *Xff lesA* gene under the control of its own promoter for an *in-planta* virulence assay (Fig. 8). When inoculated into walnut plants, which are highly susceptible to this pathogen, the infection is usually localized to a region of tumor formation at the site of infection; however, *Agrobacterium* harboring *Xff* LesA displayed a systemic necrotic response. The first signs of necrosis appeared in walnut plants inoculated with Agro-A281-LesA at eight weeks post inoculation, but not in plants that were mock-inoculated (PBS) or those that contained an empty vector (Fig. 8A,B). Interestingly, walnut plants inoculated with *Agrobacterium* expressing *Xff* LesA were dead at 12 weeks post inoculation (Fig. 8C). When inoculated into grapevines, the same phenotype was not observed (data not shown).

## Discussion

PD symptom development has long been thought to result from blockage of xylem vessels by *Xf* biofilm and associated gels and tyloses, which leads to water stress in the distal parts of the infected plant and to PD^2^. Our results support an alternative mechanism independent of water stress: a phytotoxic effect resulting from the action of a *Xff* enzyme, the LesA lipase/esterase encoded by the PD1703 locus. *Xff* LesA is similar to the type II secreted protein LipA, present in all sequenced xanthomonad genomes, and in many other Gram-negative bacteria. LipA from the rice pathogen *Xanthomonas oryzae* pv. *oryzae* (*Xoo*) is a 42 kDa α/β hydrolase fold protein with lipase/esterase function and short-chain specificity. Recently, LipA from *Xoo* was characterized as a cell wall-degrading enzyme with a carbohydrate-binding domain essential to the protein’s virulence function^21^. In *Xanthomonas campestris* pv. *vesicatoria* (*Xcv*), LipA is expressed from an early stage of tomato leaf infection and is required for wild-type virulence^22^. Additionally, treatment with *Xoo* LipA elicited callose deposition and programmed cell death in rice leaves and roots and an active site serine-to-alanine substitution reduced but did not completely suppress virulence^21^.

Is the *Xff* lipase/esterase secretion associated with the PD pathogenic process? The preponderance of evidence seems to support this hypothesis. The SSP profile identified LesA as the most abundantly secreted protein. The *Xff* SSP preparations cleaved *p*-nitrophenol butyrate and tributyrin, but no long chain triacylglycerides (Fig. S2A,B), identical to the action of the type II secreted lipase LipA. The short-chain specificity of LesA indicates that this enzyme has both esterase and lipase activities.

An association is suggested not only by the presence of homologs of PD1703 in *Xoo-* and *Xcv-*infected rice^21^,^31^,^36^ and tomato^22^, respectively, but also by its absence from *Xf* strain EB92-1, found in elderberry. EB92-1 infects and survives in grapevines for many years but does not cause symptoms and provides effective biocontrol against *Xff*. A genome draft of the EB92-1 strain revealed that 10 potential pathogenicity effectors were missing, including LesA (PD1703) and another predicted type II secreted enzyme present in our SSP profile, the serine protease PD0956^37^,^38^. On the other hand, lipase/esterase genes of *Xff* are present in the genome of an *Xf* virulent strain responsible for citrus variegated chlorosis (CVC). The CVC 9a5c strain genes XF0357, XF0358 and XF2151 are apparent homologs of the *Xff* Temecula1 genes PD1703 (LesA), PD1702 and PD1211, respectively. In a previous study, the corresponding CVC proteins were not reported in the *Xff* 9a5c secretome, possibly because the secreted protein fraction was underrepresented due to depletion by the cell washing procedure used^16^,^39^. We cannot eliminate the possibility of different secreted virulence factors among various *Xf* subspecies; however, LesA could also play a role in *Xff* 9a5c pathogenesis.

Our proteomic analysis (Table 3) revealed that LesA is one of six *Xff* proteins identified in infected grapevine leaves, clearly indicating that LesA is abundantly secreted by *Xff* during plant infection/colonization in addition to in liquid culture (Tables 1,2). If LesA is partially responsible for PD symptoms, there should be a spatial association of LesA with the leaf symptom pattern of radial transition from green tissue around the point of petiole attachment to scorching that begins at the leaf margins (Fig. 4A). Previous findings^32^ showed a gradual decrease in *Xff* titer proceeding from the leaf blade center to the leaf margin, counter to the pattern of symptom development. We have demonstrated that on a per-*Xff*-cell basis, LesA accumulates most abundantly near the leaf margins in a distribution positively correlated with the development of scorch symptoms (Fig. 4D).

As a direct demonstration of the ability of LesA to induce symptoms, LesA was produced in *E. coli* and a crude extract was pressure-infiltrated into grapevine leaf blade, inducing local necrosis. The empty-vector control did not induce necrosis and the catalytic triad control, S200A-LesA, induced only minute regions of necrosis (Fig. 5), showing further similarity to *Xoo* LipA^21^. The weak reaction of grapevine leaf to the extract from *E. coli* expressing *Xff* S200A-LesA may result from residual lipase/esterase activity. Another serine esterase retained a few percent of the wild-type activity in an active site serine-to-alanine replacement mutant^40^.

The co-localization of LesA and leaf marginal necrosis at a distance from the central leaf region of greatest *Xff* cell accumulation raised questions about LesA secretion and translocation. Two broad hypotheses were considered: i) secretion and transport of LesA as a soluble protein, or ii) entrapment of LesA in OMVs for transport toward the leaf margins. OMVs are small spherical structures that allow interaction of Gram-negative bacteria with their environment by releasing the vesicular contents adjacent to prokaryotic and eukaryotic cells. OMVs are a vehicle by which Gram-negative pathogens communicate with and intoxicate host cells^24^. The first hypothesis is fragile because if LesA is a phytotoxin, necrotic lesions should occur everywhere, including in proximity to bacterial aggregates in the central part of the leaf. But necrosis appears first at the leaf margins, favoring the second hypothesis of OMV-facilitated transport of LesA toward the leaf margin with subsequent release of the OMV cargo.

OMVs are formed by outer membrane (OM) entrapment of periplasmic constituents as OMV cargo, including soluble proteins, phospholipids, lipopolysaccharides (LPSs), and DNA^23^,^41^,^42^. Many pathogenic bacteria use OMVs to release virulence factors^43^,^44^,^45^,^46^,^47^, quorum sensing signals^48^, pathogen-associated molecular patterns (PAMPs)^49^,^50^ and other OM components like surface-exposed adhesins^51^. *Xff* releases OMVs carrying the autotransporter XatA^25^. The complete OMV protein cargo was not determined here. XatA impacts migration, colonization and biofilm formation, but a direct relationship between PD symptoms and XatA was not observed^25^.

The presence of LesA was highly concentrated in the leaf margins and its concentration gradually decreased toward the petiole, where the bacteria are typically found as a biofilm network. SEM and TEM imaging (Fig. 3) identified a major filamentous network formed in the presence of LesA, but at low concentration and well distributed. Biofilm behavior has been well studied in *Xff*^2^. Biofilms represent mainly dead cells and are down regulated for genes that are associated with disease. In contrast, the planktonic bioform is motile, spreads long distances, and expresses genes associated with disease^2^. Biofilm behavior and its relation to PD have been studied using two specific mutations, *rpfF* and *rpfC*. The *rpfF* gene is responsible for synthesis of diffusible signal factor (DSF). DSF is an unsaturated fatty acid that modulates *Xff* virulence and biofilm formation by cell–cell signaling. *Xff rpfF* mutants are deficient in DSF production, possess a hypervirulent phenotype, and are found mainly in a planktonic bioform^30^. *Xff rpfC* mutants exhibit a hyperattachment phenotype (increased biofilm formation) associated with their inability to migrate in xylem vessels and to cause PD^35^. Interestingly, gene expression analysis of *lesA* in both *rpfF* and *rpfC* quorum-sensing mutants found that *lesA* is highly down regulated in the non-pathogenic *rpfC* mutant (Fig. 7). We hypothesize that *lesA* provides specific nutrients needed to sustain planktonic growth through its cell wall degrading lipase/esterase activity, much like that observed for the pectin degrading activity of *pglA*^17^. As a consequence, *lesA* mutants that do not make LesA display a biofilm mode of growth similar to *rpfC* mutants. We believe that the inability of *Xff rpfC* mutants to cause PD symptoms in grapevine may be related in part to decreased expression of LesA and other potential virulence factors regulated by *rpfC*, as observed in *Xcc rpfC* mutants. *Xcc rpfC* mutants are deficient in the production of virulence factors such as extracellular polysaccharide (EPS) and in the secretion of extracellular enzymes^52^. Formation of OMVs by *Xff* is significantly higher in the *rpfF* mutant, which is disrupted for DSF^53^. This hypervirulent mutant also displayed XadA on the OMV’s surface, correlating with our proteomic data showing XadA abundantly present in *Xff* OMVs (Table 2). If LesA is delivered by OMVs, then increased OMV production may be partially responsible for increased *rpfF* mutant virulence. We propose that the release of LesA through OMVs to distant sites, without biofilm formation, may be a major cause of tissue destruction and result in relocation of nutrients from the leaf margins toward bacterial colonization sites.

Further corroboration of LesA participation in *Xff* pathogenesis has been shown through the two-component system of GacS/GacA. This system is involved in environmental signaling and control of secondary metabolites and production of extracellular enzymes. It regulates the virulence of many pathogenic and environmental bacteria, including *Xff*^54^. GacA is a response regulator that controls various physiological processes and pathogenicity factors through transcriptional activation of genes that regulate pathogenesis, e.g., genes involved in quorum-sensing, toxin production, motility, biofilm formation, and extracellular polysaccharide production^55^,^56^,^57^,^58^,^59^. Several putative pathogenicity-related genes are regulated by *gacA* in *Xff*, including the secreted lipase-esterase LesA. Interestingly, *Xff gacA* mutants expressed less LesA and developed significantly less severe disease symptoms when inoculated into grapevines^54^. *Xff gacA* mutants may be deficient in pathogenesis due to low expression of virulence factors such as LesA.

To confirm the correlation between LesA and *Xff* pathogenicity, we generated a *lesA* mutant, which induced less severe PD symptoms than the wild-type strain. The lack of virulence of our *Xff lesA* mutant, similar to those of *Xff rpfC, gacA, Xoo,* and *Xcv lipA* mutants^21^,^22^, supports a significant role for LesA in *Xff* pathogenicity. In addition, expression of *lesA* under its own promoter in *Agrobacterium tumefaciens* propagated a systemic cell death response in inoculated walnut plants. These findings strongly suggest that LesA is part of the *Xff* repertoire of secreted virulence factors and is directly associated with bacterial pathogenesis.

We propose a model for *Xf* pathogenesis in PD based on the secretion, movement and accumulation of the lipase/esterase LesA in infected grapevine leaves, leading to leaf scorching and chlorosis. In our preferred symptom-inducing mechanism, planktonic forms of *Xff* secrete LesA by means of OMVs. These vesicles increasingly accumulate in the leaf margins and release their cargo, including LesA, initiating PD symptom development.

## Material and Methods

### *Xff* strains and growth conditions

The WT Temecula1 strain of *Xylella fastidiosa* subspecies *fastidiosa* (*Xff*; ATCC 700964) and *Xff lesA* mutant were grown in PD3 medium^60^ with aeration (120 rpm) at 28 °C. Plate cultures were prepared in the same medium with the addition of 1.5% agar. PD3 medium was supplemented with kanamycin (5 μg/mL) for selective growth of the *lesA* mutant. The *rpfF* and *rpfC* strains used in this study were kindly provided by Prof. Steven E. Lindow.

### Isolation of secreted proteins and outer membrane vesicles from culture supernatants

*Xff* cells were harvested by centrifugation at 8000 × *g* for 15 min at 4 °C. The culture supernatant was transferred to 38.5 mL tubes and centrifuged at 38,000 × *g* for 1 h at 4 °C in a SW28 rotor (Beckman Coulter, USA). The supernatant was collected (the remaining pellet was discarded), transferred to 12 mL tubes and centrifuged at 150,000 × *g* for 3 h at 4 °C (SW41 Ti rotor, Beckman Coulter). The supernatant, containing SSPs, was concentrated 75 to 100× using Amicon Ultra-15 3 K filters units (Millipore). The pellet, containing OMVs, was resuspended in 300 μL PBS (pH 7.4) for subsequent SDS-PAGE analysis. For electron microscopy analysis, the vesicle pellet was resuspended in 50 mM HEPES buffer (pH 6.8).

### Outer membrane and total protein extraction

Outer membrane protein extraction was performed as described^27^. To prepare total protein extracts, bacteria from a 2 mL culture (four to six days old) were harvested by centrifugation at 8000 × *g* for 15 min at 4 °C and washed three times with 1 mL washing buffer containing 10 mM Tris-HCl (pH 8.8), 3 mM KCl, 50 mM NaCl, 5 mM EDTA and 1 mM PMSF and were centrifuged for 2 min at 3000 × *g*. The pelleted cells were then lysed with 200 μL 10 mM Tris (pH 8.8) with 0.5% w/v SDS, 5 mM EDTA, and 1 mM PMSF. After adding DTT to 100 mM, the sample was boiled 3 min and stored at −80 °C.

### Bacterial surface digestion

*Xff* cells were harvested by centrifugation at 8000 × *g* for 15 min at 4 °C and washed three times with 1 mL PBS. A total of 4 × 10^8^ cells were resuspended in 100 μL filtered and sterilized 50 mM ammonium carbonate buffer (pH 7.5). Digestions were carried out with 20 μg sequencing grade modified trypsin (Promega) in the presence of 5 mM DTT for 15 min at 37 °C. The digestion mixture was centrifuged at 3500 × *g* for 10 min at 4 °C and the supernatant (containing the peptides) was collected. The trypsin reaction was stopped by adding formic acid to 0.1%. The reaction was filtered using Amicon Ultra Microcon 3 K filters units (Millipore), and the flow-through peptides were held at −20 °C for later analysis.

### Grapevine leaf protein extraction

Grapevine (*Vitis vinifera* L. cv. ‘Thompson Seedless’) leaves from *Xff*-infected (n = 5) and non-infected (n = 5) plants were collected about one meter above the point of inoculation (12 wpi). Plant tissues were flash frozen in liquid nitrogen, lyophilized and kept at -80°C. Proteins were extracted using a phenol extraction procedure as described^61^. Each leaf segment (Fig. 4A) was ground in liquid nitrogen using a pestle and mortar containing 1% (w/w) PVPP. One hundred mg plant material was resuspended in 600 μL extraction buffer (0.7 M sucrose, 0.1 M KCl, 0.5 M Tris-HCl pH7.5, 0.5 M EDTA, 1 mM PMSF and 2% β-mercaptoethanol). The suspension was homogenized three times (1 min each) using a MM300 TissueLyser (Qiagen). An equal volume of UltraPure buffer-saturated phenol (Life Technologies, USA) was added and the mixture was rehomogenized as described above. After centrifugation at 12,000 × *g* for 15 min at 4°C, the upper phenol phase was removed and the remaining pellet used for re-extraction in extraction buffer. Proteins were precipitated from the phenol phase using five volumes of 100 mM ammonium acetate in methanol overnight at -20°C followed by centrifugation at 12,000 × *g* for 15 min at 4°C. Protein pellets were washed four times with 4 mL 100 mM ammonium acetate in methanol and dried 10 min in the hood. Proteins were solubilized with urea buffer (7 M urea, 2 M thiourea, 40 mM Tris, 2% Chaps and 18 mM DTT). The protein concentration was determined according to Bradford’s method using BSA as the standard.

### Protein preparation and mass spectrometry analysis

*Xff* SSPs and OMV proteins were resolved by SDS-PAGE prior to in-gel digestion to reduce the amount of non-protein contaminants in the samples. Peptides from the cell shaving (surfaceome) procedure were desalted using Aspire RP30 desalting tips (Thermo-Fisher Scientific) and subjected directly to LC/MSMS analysis. For the in-gel digestion used for SSPs and OMV proteome analysis, gel pieces were washed twice with 100 to 150 μL 50 mM ammonium bicarbonate (AmBic; pH 8.0), followed by dehydration with acetonitrile (ACN; three to four times the total volume of gel pieces) for 10 to 15 min. Proteins were reduced for 30 min at 56°C in a solution of 10 mM DTT and 50 mM AmBic. Gel pieces were dehydrated again, followed by replacement of ACN by 55 mM iodoacetamide in 50 mM AmBic. Gel pieces were incubated 20 min in the dark at room temperature, followed by two washes with 150 to 200 μL of 50 mM AmBic for 15 min each. Gel pieces were dehydrated with ACN, dried by speed vacuum centrifugation and subjected to tryptic digestion overnight. Peptides were extracted by adding 60% ACN and 0.1% trifluoroacetic acid (TFA) in water to the gel pieces, followed by sonication for 10 min. The solution containing the peptides was mixed with the supernatant resulting from the tryptic digestion, followed by speed vacuum centrifugation. Digested peptides were then desalted using Aspire RP30 desalting tips and resuspended in loading buffer.

The digested peptides were analyzed using a QExactive mass spectrometer (Thermo Fisher Scientific) coupled with an Easy-LC (Thermo Fisher Scientific) and a nanospray ionization source. The peptides were loaded onto a trap (100 micron, C18 100 Å 5U) and desalted online before separation using a reverse phased column (75 micron, C18 200 Å 3U). The gradient duration for separation of peptides was 60 min using 0.1% formic acid and 100% ACN for solvents A and B respectively. Data was acquired using a data-dependent ms/ms method with a full scan range of 300 to 1600 Da and a resolution of 70,000. The ms/ms method’s resolution was 17,500 with an isolation width of 2 m/z with normalized collision energy of 27. The nanospray source was operated using 2.2 KV spray voltage and a heated transfer capillary temperature of 250°C. Raw data was analyzed using X!Tandem and visualized using Scaffold Proteome Software (Version 3.01). Samples were searched against Uniprot databases appended with the cRAP database, which recognizes common laboratory contaminants. Reverse decoy databases were also applied to the database prior to the X!Tandem searches.

For the grapevine proteomic analysis, leaf proteins were precipitated using a ProteoExtract protein precipitation kit (Calbiochem) followed by dehydration overnight in a sterile fume hood. The protein pellet was resuspended in 50 mM AmBic (pH 8.0) and subjected to an in-solution tryptic digestion. Digested peptides were then desalted and subjected to LC/MSMS as described above.

### Electron microscopy analysis of outer membrane vesicles

Outer membrane vesicles were resuspended in 50 mM HEPES buffer (pH 6.8) and fixed with 4% paraformaldehyde in 1 M Sorenson’s phosphate buffer (pH 7.4). Copper grids (400 mesh) supported with formvar coating were used for electron microscopy. Ten μL fixed OMVs were placed in the grids and allowed to settle for 10 min. The excess sample was removed with filter paper, followed by quickly staining with 1% ammonium molybdate. Grids were air-dried completely before visualization in a Philips CM120 (FEI/Philips Inc.) electron microscope at 80 KV.

### Western-blot analysis

To detect LesA, anti-LesA antibody was diluted in PBS-M 1% (PBS plus 1% non-fat dried milk; 1:1000), followed by detection using HRP-conjugated goat anti-rabbit antibody (1:2000 for grapevines and 1:4000 for *Xff* samples) (Life Technologies, USA). Blocking and washing steps were performed with PBS-M 5% (PBS plus 5% non-fat dried milk) and PBS-T 0.1% (PBS plus 0.1% Tween 20), respectively. Developments were carried out using ECL Plus western blotting detection reagents (GE Life Sciences, USA). To detect MopB and EF-Tu in *Xff* samples, polyclonal antibodies (dilutions of 1:20,000 for MopB and 1:10,000 for EF-Tu) were used, followed by detection using HRP-conjugated goat anti-rabbit antibody (1:20,000). Blocking and washing steps were carried out as described for LesA. Blots were developed using SuperSignal West Dura Chemiluminescent Substrate (Thermo Scientific, USA). The anti-LesA polyclonal antibody was generated by immunizing rabbits with LesA immunogenic synthetic peptides (GeneScript, USA). The anti-MopB^27^ and anti-EF-Tu polyclonal antibodies were kindly provided by George Bruening (Plant Pathology, UC Davis).

### Immunogold electron microscopy

Immunogold electron microscopy (IEM) was performed using fresh cultures of *Xff*. The sample containing *Xff* cells and the filamentous network secreted in the culture supernatant were fixed with 4% paraformaldehyde in 1 M Sorenson’s phosphate buffer (pH 7.4). The fixed sample was embedded in LR White resin as described^62^. Ultra-thin sections were cut and placed onto coated grids (200 mesh; treated with glow-discharge), followed by blocking with 1% fish gelatin for 30 min. Grids were blotted with anti-LesA (1:500) antibody for 1 h at RT and washed with PBS. The primary antibody was detected using anti-rabbit (1:50) antibody coupled to 10 nm gold particles. Unbound conjugate was removed using a sequence of washing steps with PBS. The preparation was negatively stained with 1% ammonium molybdate, air-dried, and visualized in a Philips CM120 (FEI/Philips Inc.) electron microscope at 80 KV.

### Lipase and esterase activity assays

Tributyrin (C4), tricaprin (C10), and a mixture of tryglicerides (C2 to C10) (Sigma-Aldrich, USA) were used as substrates for LesA activity in a plate assay^21^,^63^. The triglyceride substrates (0.5%; v/v) were prepared in a buffer containing 100 mM Tris-HCl (pH 8.0), 25 mM CaCl_2_, sonicated at 30 W for three min to emulsify the substrates, mixed with an equal volume of 2% agarose solution and solidified in Petri plates. Fifty μL *Xff* soluble supernatant proteins (700 ng/μL) were added to the wells and assayed for a zone of clearance for 24 to 48 h at RT. The culture medium PD3 was used as a negative control. For the *Xff lesA* mutant lipase activity assay, tributyrin (3%; v/v) was emulsified in PD3 agar medium using a Polytron PT 3100 prior to autoclaving. Esterase activity was also determined using 4-methylumbelliferyl butyrate (4-MUB) substrate as previously described^64^. For the SSPs esterase activity assay, pNP butyrate (pNP-C4) was used as the substrate in a spectrophotometric assay, which was performed at A_405_ after a 10 min incubation of pNP-C4 (1 to 5 mM) with *Xff* soluble supernatant proteins (1 μg/well) in 50 mM Tris-HCl (pH 7.5) at 37 °C.

### LesA detection in grapevine leaves by ELISA

To detect LesA in grapevine (*Vitis vinifera* L. c.v. ‘Thompson Seedless’) by ELISA, leaves from *Xff*-infected (12 wpi) and non-infected plants were collected and divided in three sections (inside, middle and outside; ∼2 cm radial dimension each). Ninety mg leaf tissue was homogenized in 900 μL coating buffer (0.1 M sodium carbonate buffer, pH 9.6) for three min using a MM300 TissueLyser (Qiagen, USA). The number of *Xff* cells present in each homogenized sample was determined using a double-antibody sandwich (DAS)-ELISA assay (Agdia Inc., USA) following manufacturer’s instructions. For LesA detection, the homogenized solution was used to coat (100 μL/well) a ninety-six-well Maxisorp microtiter plate (NUNC, USA) for two h at RT. The wells were washed two times with PBS-T 0.1% (PBS plus 0.1% Tween 20) and blocked for one h at RT with PBS-M 5%. The plate was washed three times with PBS-T 0.1%, followed by incubation with anti-LesA (1:1000) antibody in PBS-M 1% for one h at 37 °C. The plate was washed three times with PBS-T 0.1% followed by incubation with HRP-conjugated anti-rabbit (1:1000) in PBS-M 1% for one h at 37 °C. The plate was washed four times with PBS-T 0.1% and developed with TMB (3,3′,5,5′-tetramethylbenzidine). One-way ANOVA and Mann-Whitney tests were carried out using GraphPad Prism 5.0 (GraphPad Software Inc., San Diego, CA). A value of p < 0.05 was considered statistically significant.

### Hypersensitive response assay in grapevine leaves

The heterologous expression of wild type LesA and its mutated version, S200A-LesA, in which the amino acid Ser-200 of the active triad was replaced by alanine, was accessed after cloning both genes in the pJexpress 401 (DNA2.0, USA) vector for expression under the T5 promoter. The insertion of cloned genes was verified by PCR using primers for sites flanking LesA genes, followed by transformation of ElectroMAX DH5α competent cells (Life Technologies, USA). For heterologous expression, cells were grown in LB medium supplemented with kanamycin (50 μg/mL) at 37 °C and 120 rpm to an A_600_ of 0.8. IPTG (1 mM) was added to the culture, followed by incubation for three h at 30 °C and 120 rpm. Total protein was extracted from *E. coli* cells using a CellLytic B Plus dit (Sigma-Aldrich, USA). The protein concentration was determined according to Bradford’s method using BSA as the standard. Total proteins from LesA, S200A-LesA, and empty vector-expressing *E. coli* were used for syringe infiltration in greenhouse-grown grapevine (*Vitis vinifera* L. c.v. ‘Thompson Seedless’) leaves. One hundred μg/spot of protein (1 μg/μL) was infiltrated into each leaf spot. HR-like lesions in the infiltrated area were photographed 24 h after inoculation.

### Isolation of the *lesA* mutant strain

The mutagenesis cassette was chemically synthesized (GenScript, USA) after insertion of a kanamycin resistance gene within Ser-200, the first amino acid of the active triad. The entire open reading frame of PD1703 (494 bp at 5′ and 670 bp at 3′) was chosen as flanking homology regions in this cassette. The synthesized pUC57-PD1703::kan was electroporated into *Xff* WT Temecula1 as described previously^65^. Primers outside of PD1703 were used to confirm double crossover events in transformants using gel electrophoresis and sequencing.

### Grapevine infection and disease quantification

Grapevines (‘Thompson Seedless’) were inoculated with 20 μL *Xff* suspension containing ∼2 × 10^7^ cells. The plants were inoculated with 10 μL on the first day and reinoculated with 10 μL on the second day, with an independently grown *Xff* culture used for each inoculation. The bacteria were introduced into each plant at three to four inches above the soil using a number 0 insect pin. Twenty-five plants were inoculated with *Xff* wild type strain, 26 plants with *lesA* mutant, and 26 plants with PBS (mock inoculation). Quantification of symptoms was performed using 18 randomly selected plants/treatment at 14 wpi. We show the average percent scorching of the vine with standard error, with nodes without leaves and petioles being excluded from this analysis. Leaf Point System: 0-24% scorching of leaf = 0 points, 25-49% = 1 pt, 50-74% = 2 pts, 75-100% = 3 pts. Values for individual leaves were then summed and divided by the total possible scorching (3 * number of leaves) to give one value per vine. One-Tailed T-test with Welch’s correction was carried out using GraphPad Prism 5.0 (GraphPad Software Inc., San Diego, CA). A value of p < 0.05 was considered statistically significant.

### RNA extraction and real-time RT-PCR

*Xff* 3A2 (WT), *rpfC* and *rpfF* cells were grown in 50 mL PD3 liquid medium (three flasks/condition) until the cultures reached 10^7^ to 10^8^ cells/mL. RNA was extracted using the miRNeasy Mini Kit (Qiagen, USA) following manufacture’s instructions. cDNA was synthesized using SuperScript III First-Strand Synthesis SuperMix (Life Technologies, USA). RT-PCR reactions were performed using TaqMan Universal PCR Master Mix (Life Technologies, USA) on the StepOne Real-Time PCR System (PE Applied Biosystems, USA). The level of gene transcription was normalized to 16S rRNA and expressed as a relative difference. The statistical analysis was performed using GraphPad Prism software, version 5 (Graph-Pad Software, San Diego, USA), using the Unpaired t test with Welch’s correction. The data was considered significant when p < 0.05.

### *Agrobacterium* virulence assay in walnut plants

BPROM, a bacterial sigma70 promoter recognition program, was used to predict the potential promoter for *lesA*. This software predicted one promoter region starting at 229 bps 5′ of the *lesA* start codon. The *lesA* open reading frame and the 500 bps 5′ of the intergenic space, which was predicted to include the promoter region for *lesA*, were cloned into the binary vector pDU97.1005. Expression of LesA was tested by detecting protein activity on a tributyrin agar plate. The presence of LesA activity ensured that the predicted 500 bps 5′ of this gene contain the active *lesA* promoter region, since no other promoter was present in the binary vector. Subsequently, positive colonies with LesA activity were transformed into a disarmed *Agrobacterium* strain (EHA101-PCH32) by electroporation. The *lesA* region and empty vector containing *Agrobacterium* strains were infected into walnut (Chandler) by creating an incision in the bark on the main stem. Ten μL resuspended bacterial culture (10^8^ cells in PBS) was placed on the incision and wrapped with parafilm. The symptoms were observed at eight and 12 weeks post inoculation.

## Additional Information

**How to cite this article**: Nascimento, R. *et al.* The Type II Secreted Lipase/Esterase LesA is a Key Virulence Factor Required for *Xylella fastidiosa* Pathogenesis in Grapevines. *Sci. Rep.* **6**, 18598; doi: 10.1038/srep18598 (2016).

## Supplementary Material

Supplementary Information

## Figures and Tables

**Table 1:** *X. fastidiosa* Temecula 1 soluble supernatant proteins (SSPs) identified in the secretome.

Accessionnumber^a^	Protein description	Genename	OMV	Surface	OM	Prot.local.^b^	Theor.Mw^c^	Signalpeptide^d^	SecPscore^e^	Seq.cover	Matchedpeptides
Q87AW0	Putat. unchar. protein	PD1703	√	√	—	U	42.4	—	0.889430	78.8%	102
Q87AW1	Putat. unchar. protein	PD1702	√	—	—	U	42.7	—	0.885983	83.9%	36
Q87DF4	Outer memb. protein XadA	PD0731	√	√	—	U	97.5	—	0.963014	34.8%	28
Q87BM1	Bacteriocin	PD1427	√	√	—	EC	150.3	—	0.605475	19.2%	21
Q87EJ4	Serine protease	PD0313	√	—	—	OM	101.3	26|27	—	22.1%	20
Q87DM5	Putat. unchar. protein	PD0657	—	—	—	EC	34.1	29|30	—	37.0%	7
Q87C82	Putat. unchar. protein	PD1211	√	—	—	U	46.4	—	0.939004	30.2%	13
Q87AL6	Outer memb. prot. OmpW	PD1807	√	—	√	OM	23.1	23|24	—	36.7%	12
Q87AV4	Outer memb. prot. MopB	PD1709	√	√	√	OM	42.3	—	0.939696	24.4%	7
Q87BF0	Hemolysin-type Ca-binding prot.	PD1506	√	—	—	EC	164.2	—	0.728400	7.23%	5
Q87CV6	Serine protease	PD0950	—	—	—	OM	96.0	—	0.914469	7.26%	4
Q87E92	Chorismate mutase	PD0426	—	—	—	P	21.4	—	0.115485	34.0%	6
Q87CV2	Putat. Unchar. Prot.	PD0956	—	—	√	U	37.2	—	0.890737	21.6%	6
Q87BZ7	Putat. unchar. protein	PD1299	—	√	—	U	55.7	—	0.898888	11.1%	6
Q87D31	VirK protein	PD0855	—	—	—	U	16.0	22|23	—	24.8%	4
Q87AH5	Peptidase (M20/M25/M40 family)	PD1850	—	—	—	U	57.7	—	0.582143	7.94%	4
Q87EI9	Putat. unchar. protein	PD0318	—	—	√	OM	110.9	33|34	—	3.87%	4
Q87E00	Cellulose 1,4-beta-cellobiosidase	PD0529	—	—	—	OM	67.7	—	0.883389	7.31%	3
Q87AN1	Hemagglutinin-like protein	PD1792	—	—	—	OM	355.2	—	0.957381	0.95%	3
Q87BN7	Aminotransferase	PD1411	—	—	—	C	46.3	—	0.092435	3.06%	2
Q87DY9	Putat. unchar. protein	PD0540	—	√	√	U	15.2	—	0.903987	18.0%	2
Q87D30	Peptidyl-dipeptidase	PD0856	—	√	√	C	77.5	—	0.798642	2.62%	2
Q87CK4	Putat. unchar. protein	PD1063	—	—	√	OM	21.1	20|21	—	13.7%	2
Q87C13	TonB-dep. receptor	PD1283	√	—	√	OM	102.7	31|32	—	2.82%	2

^a^Protein accesion number at UniProt Knowledgebase (UniProtKB; http://www.uniprot.org/).

^b^Protein localization as prediceted by PSORTb v. 3.0.2 Subcellular Localization Prediction Tool (http://www.psort.org/psortb/; C: cytoplasmic; OM: outer membrane; P: periplasmic; EC: extracellular; U: unknown).

^c^Theoretical protein molecular weight shown in kDa.

^d^Position of signal peptide cleavage site as predicted by SignalP 4.0 Server (http://www.cbs.dtu.dk/services/SignalP/).

^e^Prediction of non-classical protein secretion by SecretomeP 2.0 Server (http://www.cbs.dtu.dk/services/SecretomeP/) used for proteins without signal peptide. Score >0.5 indicates non-classical secretion.

**Table 2:** Proteins identified in *X. fastidiosa* Temecula 1 outer membrane vesicle (OMV) proteomic analysis.

Accessionnumber^ a^	Protein description	Genename	SSP	Surface	OM	Prot.local.^b^	Theor.Mw^c^	Sequencecoverage	Matchedpeptides
Q87DF4	Outer membrane protein XadA	PD0731	√	√	—	U	97.5	55.0%	61
Q87AW0	Putative uncharacterized protein	PD1703	√	√	—	U	42.4	67.0%	22
Q87AW1	Putative uncharacterized protein	PD1702	√	—	—	U	42.7	63.0%	11
Q87AL6	Outer membrane protein ompW	PD1807	√	—	√	OM	23.1	19%	6
Q87AV4	Outer membrane protein mopB	PD1709	√	√	√	OM	42.3	15%	5
Q87AA4	Fimbrial protein	PD1924	—	√	√	EC	15.3	15%	2
Q87BM1	Bacteriocin	PD1427	√	√	—	EC	150.3	8%	7
Q87EJ4	Serine protease	PD0313	√	—	—	OM	101.3	4%	4
Q87C13	TonB-dependent receptor	PD1283	√	—	√	OM	102.7	4%	3
Q87C82	Putative uncharacterized protein	PD1211	√	—	—	U	46.4	6%	2
Q87BF0	Hemolysin-type calcium binding protein	PD1506	√	—	—	EC	164.2	4%	2

^a^Protein accesion number at UniProt Knowledgebase (UniProtKB; http://www.uniprot.org/).

^b^Protein localization as prediceted by PSORTb v. 3.0.2 Subcellular Localization Prediction Tool (http://www.psort.org/psortb/; OM: outer membrane; EC: extracellular; U: unknown).

^c^Theoretical protein molecular weight in kDa.

**Table 3:** *X. fastidiosa* Temecula 1 proteins found in infected grapevine leaf proteomic analysis.

Accessionnumber^a^	Protein description	Genename	SSP	Surface	OMV	OM	Prot.local.^b^	Theor.Mw^c^	Sequencecoverage	Matchedpeptides
Q87AW0	Putative uncharacterized protein	PD1703	√	√	√	—	U	42.4	19.6%	7
Q87AV4	Outer membrane protein MopB	PD1709	√	√	√	√	OM	42.3	11.1%	3
Q87BC0	60 kDa chaperonin	PD1538	—	√	—	√	C	57.7	6.2%	2
Q87B34	Glyceraldehyde-3-phosphate dehydrogenase	PD1626	—	—	—	√	C	36.0	5.0%	2
P63774	10 kDa chaperonin	PD1537	—	√	—	√	C	10.0	25.3%	2
Q87DE1	Surface protein	PD0744	—	—	—	—	U	203.1	1.46%	2

^a^Protein accesion number at UniProt Knowledgebase (UniProtKB; http://www.uniprot.org/).

^b^Protein localization as prediceted by PSORTb v. 3.0.2 Subcellular Localization Prediction Tool (http://www.psort.org/psortb/; C: cytoplasmic; OM: outer membrane; U: unknown).

^c^Theoretical protein molecular weight in kDa.
