# Corrigendum: The Type II Secreted Lipase/Esterase LesA is a Key Virulence Factor Required for *Xylella fastidiosa* Pathogenesis in Grapevines

**DOI:** 10.1038/srep21575

**Published:** 2016-02-25

**Authors:** Rafael Nascimento, Hossein Gouran, Sandeep Chakraborty, Hyrum W. Gillespie, Hebréia O. Almeida-Souza, Aye Tu, Basuthkar J. Rao, Paul A. Feldstein, George Bruening, Luiz R. Goulart, Abhaya M. Dandekar

Scientific Reports 6: Article number: 1859810.1038/srep18598; published online: 01122016; updated: 02252016.

The Acknowledgements section in this Article is incomplete.

“The authors acknowledge research support obtained from the Citrus Research Board and the CDFA PD Board of California. RN is grateful to Brazilian agency CAPES for scholarship’s support. We thank David Dolan for his contributions to experimental procedures in the greenhouse. We express our gratitude to Grete Adamson and Patricia Kysar (Electron Microscopy Laboratory, School of Medicine, University of California at Davis) for technical support.”

should read:

“The authors acknowledge research support obtained from the Citrus Research Board and the CDFA PD Board of California. RN is grateful to Brazilian agency CAPES for scholarship’s support. We wish to acknowledge Brett Phinney the manager of the Proteomics core facility (UC Davis Genome Center) for his skill and insight in conducting the proteomic analysis of our samples. We thank David Dolan for his contributions to experimental procedures in the greenhouse. We express our gratitude to Grete Adamson and Patricia Kysar (Electron Microscopy Laboratory, School of Medicine, University of California at Davis) for technical support.”

