# Supplementary material for: The Type II Secreted Lipase/Esterase LesA is a Key Virulence Factor Required for Xylella fastidiosa Pathogenesis in Grapevines
Source: Sci Rep. 2016 Jan 12;6:18598. doi: 10.1038/srep18598 (PMC4709584; doi:10.1038/srep18598)
Supplement: Supplementary Information [file srep18598-s1.pdf]

Supplementary Information

The Type II Secreted Lipase/Esterase LesA is a Key Virulence Factor Required for *Xylella fastidiosa* Pathogenesis in Grapevines

Rafael Nascimento, Hossein Gouran, Sandeep Chakraborty, Hyrum Gillespie, Hebréia O. Almeida-Souza, Aye Tu, Basuthkar J. Rao, Paul A. Feldstein, George Bruening, Luiz R. Goulart, and Abhaya M. Dandekar.

Supplementary Figures

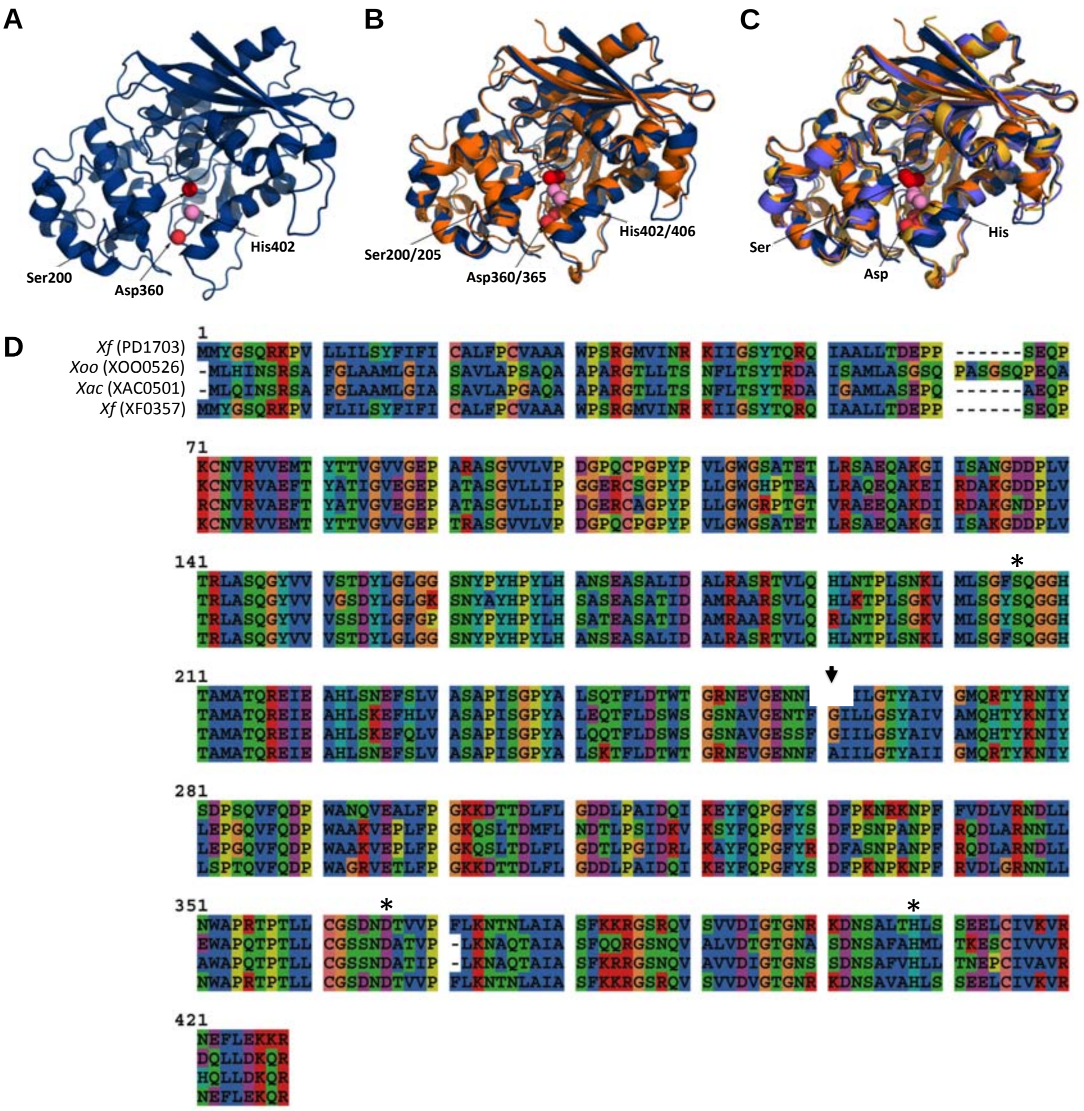

Fig. S1: *In silico* analysis of LesA. The structures for PD1703 (*X. fastidiosa* Temecula1), XF0357 (*X. fastidiosa* 9a5c) and XAC0501 (Xac, *Xanthomonas axonopodis* pv. citri) were predicted using GENO3D[1]. (**A**) The catalytic active triad in PD1703. The multiple superimpositions of these structures were obtained using MUSTANG[2]. (**B**) The alignment of 3H2G (XOO0526 from Xanthomonas oryzae pv. oryzae) and PD1703 (colored orange and blue, respectively). (**C**) The alignment of all four structures: 3H2G (XOO0526), PD1703, XF0357 and XAC0501 (colored orange, blue, slate and yellow, respectively). (**D**) The multiple sequence alignment for these proteins was generated using Clustal W[3]. The catalytic triad Ser-Asp-His is indicated by asterisks. In *Xf* strains, the residue Ala replaces the residue Gly-260 (indicated by a black arrow), which is required for the sugar ring positioning of the LipA-like carbohydrate-binding domain in *Xoo*[4]. The alignment image was generated using Seaview[5].

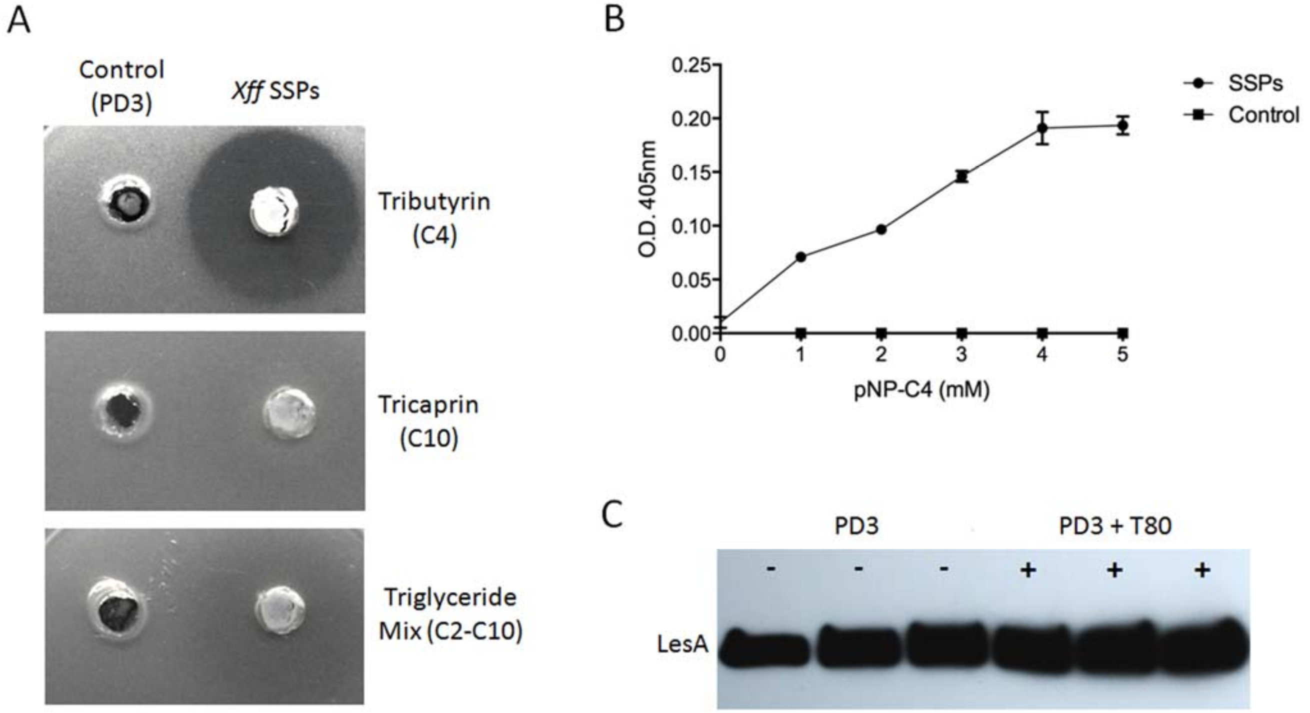

Fig. S2: *Xff* SSPs exhibit lipase/esterase activities. Triglycerides were incorporated into an agarose gel layer. Holes on the left received PD3 medium (control); holes on the right received *Xff* SSPs. (**A**) Presence of a zone of clearance indicates LesA activity on short-chain triacylglycerides (C4-Tributyrin), while no activity is seen on long-chain triacylglycerides (C10-Tricaprin). Weak activity is seen on a mixture of triglycerides that contains short- and long-chain triacylglycerides (C2-C10). (**B**) Esterase activity of *Xff* SPPs towards p-nitrophenol butyrate (pNP-C4) at 37°C in Tris-HCl, pH 7.5. A405 was measured after 10 min incubation with increasing concentrations of the substrate. (**C**) Induction *in vitro* of LesA protein expression by Tween 80 was accessed after growing *Xff* in PD3 cultures supplemented with 0.1% (v/v) Tween 80 (3 flasks/condition; growth until $3x10^8$ cells/mL) followed by LesA immunoblot detection.

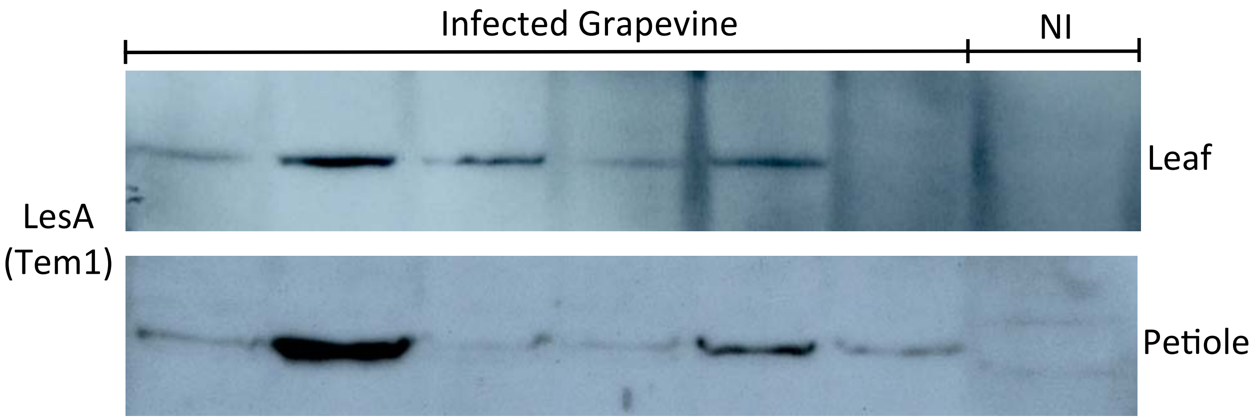

Fig. S3: Detection of LesA in grapevines infected with *Xff*. Leaf and petiole proteins extracted from *Xff*-infected (I; 12 wpi) and non-infected (NI) grapevines were used for detection of LesA by immunoblotting.

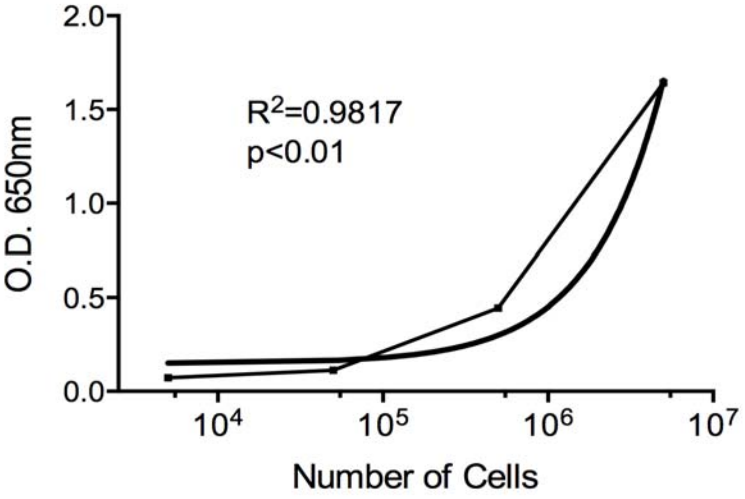

Fig. S4: Standard curve used for quantification of bacterial cells in grapevine leaf tissues by double-antibody sandwich (DAS)-ELISA assay.

Table S1: List of *X. fastidiosa* Temecula 1 proteins identified in the surfaceome (cell shaving) analysis.

Accession number[1]	Protein description	Gene name	SSP	OMV	OM	Prot. local.[2]	Theor. Mw[3]	Sequence coverage	Matched peptides
Q87DF4	Outer membrane protein XadA	PD0731	√	√	-	U	97.5	12.10%	12
Q87BD1	Peptidyl-prolyl cis-trans isomerase	PD1525	-	-	√	OM	25.0	25.40%	6
Q87AW0	Putative uncharacterized protein	PD1703	√	√	-	U	42.4	20.40%	9
Q87BC0	60 kDa chaperonin	PD1538	-	-	√	C	57.7	9.32%	6
Q87DY9	Putative uncharacterized protein	PD0540	√	-	√	U	15.2	11.60%	2
Q87A98	Succinyl-CoA ligase [ADP-forming] subunit beta	PD1930	-	-	√	C	41.0	10.60%	4
Q87BM1	Bacteriocin	PD1427	√	√	-	EC	150.3	2.55%	4
Q87BZ7	Putative uncharacterized protein	PD1299	√	-	-	U	55.7	5.73%	3
Q87A38	Temperature acclimation protein B	PD1993	-	-	√	C	8.0	38.40%	3
Q87BK3	UTP-glucose-1-phosphate uridylyltransferase	PD1450	-	-	√	C	32.6	5.74%	2
P66380	30S ribosomal protein S12	PD1999	-	-	√	C	13.6	14.50%	2
Q87A29	50S ribosomal protein L1	PD2004	-	-	√	C	24.3	9.48%	2
Q87D30	Peptidyl-dipeptidase	PD0856	√	-	√	C	77.6	4.51%	3
Q87AA4	Fimbrial protein	PD1924	-	√	√	EC	15.3	7.43%	2
P66349	30S ribosomal protein S10	PD0436	-	-	√	C	11.5	10.70%	2
Q87D28	Putative uncharacterized protein	PD0858	-	-	-	U	18.5	12.20%	2
P66605	30S ribosomal protein S6	PD1945	-	-	√	C	16.4	6.29%	1
P63774	10 kDa chaperonin	PD1537	-	-	√	C	10.0	15.80%	2
Q87ER8	Putative uncharacterized protein	PD0230	-	-	√	U	12.5	9.09%	1
Q87A33	DNA-directed RNA polymerase subunit beta'	PD2000	-	-	√	C	155.8	1.56%	2
Q87A00	Oxidoreductase	PD2039	-	-	√	U	80.5	2.08%	2
Q87A31	50S ribosomal protein L7/L12	PD2002	-	-	√	U	12.7	14.80%	2
Q87AV4	Outer membrane protein MopB	PD1709	√	√	√	OM	42.3	3.08%	1
Q877P8	Elongation factor Tu	PD1996	-	-	√	C	42.9	1.52%	1
Q87CB5	Peptidyl-prolyl cis-trans isomerase	PD1168	-	-	√	OM	24.8	6.90%	1
Q87E60	30S ribosomal protein S4	PD0460	-	-	√	C	23.4	4.33%	1
Q87E05	Phosphoenolpyruvate synthase	PD0524	-	-	√	C	87.3	1.00%	1

1: Protein accesion number at UniProt Knowledgebase (UniProtKB; http://www.uniprot.org/);
2: Protein localization as prediceted by PSORTb v. 3.0.2 Subcellular Localization Prediction Tool (http://www.psort.org/psortb/; C: Cytoplasmic; OM: Outer Membrane; EC: Extracellular; U: Unknown);
3: Theoretical protein molecular weight shown in kDa.

Table S2: List of *X. fastidiosa* Temecula 1 proteins identified in the outer membrane proteomic analysis.

Accession number[1]	Protein description	Gene name	Prot. local.[2]	Theor. Mw[3]	Sequence coverage	Matched peptides
28199417	60 kDa chaperonin	groL	C	58	97.8	66
28199862	Elongation factor Tu	tufA	C	43	86.1	32
28199584	Outer membrane protein	mopB	OM	42	49.3	25
28199254	Chaperone protein	dnaK	C	68	58.3	32
28198761	Peptidyl-dipeptidase	dcp	C	78	48.7	26
28199866	DNA-directed RNA polymerase subunit beta'	rpoC	C	156	41.3	41
28199169	TonB-dependent receptor	PD1283	OM	103	42.9	27
28199863	Elongation factor G	fusA	C	78	44.2	21
28199337	30S ribosomal protein S1	rpsA	C	62	46.2	17
28199830	Outer membrane export factor	tolC	OM	49	55.5	20
28199867	DNA-directed RNA polymerase subunit beta	rpoB	C	155	24.5	20
28198797	Outer membrane protein P6 precursor	PD0895	OM	21	44.3	11
28198239	Putative uncharacterized protein	PD0318	OM	111	23.0	18
28198193	Polyphosphate-selective porin O	oprO	OM	44	41.6	15
28198133	ATP-dependent RNA helicase	deaD	C	68	43.0	16
28198042	Leucyl aminopeptidase	pepA	C	52	43.1	13
28199166	Low molecular weight heat shock protein	hspA	U	18	73.7	9
28198164	Bifunctional aconitate hydratase 2	acnB	C	94	27.5	16
28199416	10 kDa chaperonin	groS	C	10	85.2	10
28199827	30S ribosomal protein S2	rpsB	C	29	50.0	11
28198356	50S ribosomal protein L2	rplB	C	30	41.8	10
28199449	Metallopeptidase	PD1570	P	78	32.7	14
28198247	Outer membrane antigen	oma	OM	88	21.0	13
28199468	TonB-dependent receptor	btuB	OM	114	31.9	17
28198925	Putative uncharacterized protein	PD1028	U	25	51.8	7
28199864	30S ribosomal protein S7	rpsG	C	17	49.6	13
28198126	Polynucleotide phosphorylase/polyadenylase	pnp	C	76	24.8	14
28199507	HflK protein	hflk	U	41	45.1	11
28198196	Heat shock protein 90	htpG	C	72	31.7	12
28199868	50S ribosomal protein L7/L12	rplL	U	13	57.3	11
28198158	Putative uncharacterized protein	PD0230	U	13	91.7	9
28198376	30S ribosomal protein S4	rpsD	C	23	58.6	10

28198666	Alpha-ketoglutarate decarboxylase	odhA	C	105	23.3	13
28198122	Translation initiation factor IF-2	infB	C	97	30.6	19
28199900	Oxidoreductase	PD2039	U	81	24.0	11
28198665	Dihydrolipoamide acetyltransferase	sucB	C	42	28.9	10
28199714	Fructose-bisphosphate aldolase	PD1845	C	36	41.6	8
28198359	30S ribosomal protein S3	rpsC	C	27	49.0	10
28199904	Protease IV	sppA	CM	69	23.5	10
28199497	Serine/threonine protein kinase	PD1620	OM	43	40.5	9
28198377	DNA-directed RNA polymerase subunit alpha	rpoA	C	37	44.3	13
28199574	OmpA family protein	ompA	OM	26	61.2	11
28198344	ATP synthase subunit beta	atpD	C	51	43.8	13
28198365	50S ribosomal protein L5	rplE	C	20	58.1	10
28199791	Fimbrial protein	pilA	EC	15	78.4	9
28199622	Putative ABC transporter ATP-binding protein	PD1751	C	62	24.5	11
77747677	Ketol-acid reductoisomerase	ilvC	C	39	46.6	10
28199859	Temperature acclimation protein B	PD1993	C	8	87.7	6
28199798	Succinyl-CoA synthetase subunit alpha	sucD	C	30	62.8	11
28198651	Subunit C of alkyl hydroperoxide reductase	ahpC	C	21	53.2	8
28198689	Precursor of drug resistance protein	acrA	U	44	41.7	9
28199797	Succinyl-CoA ligase [ADP-forming] subunit beta	sucC	C	41	29.7	8
28198385	Trigger factor	tig	C	48	23.0	7
28198007	Cell division protein	hflB	CM	71	17.8	6
28199726	Protein translocase subunit secA	secA	C	103	15.3	9
28199849	Ribonuclease E	rne	U	126	15.1	8
28199810	50S ribosomal protein L9	rplI	C	16	57.7	4
28198614	Virulence regulator	PD0708	C	16	61.6	5
28199748	Lipase/esterase	estA	OM	64	29.0	10
28198477	Septum site-determining protein	minD	C	29	78.1	13
28198269	Succinate dehydrogenase flavoprotein subunit	sdhA	CM	65	14.3	6
28199826	Elongation factor Ts	tsf	C	31	39.4	8
28199039	Twitching motility protein	pilT	C	38	36.0	10
28199506	Integral membrane proteinase	hflC	U	32	32.8	7
28197955	Biopolymer transport ExbB protein	exbB	CM	27	28.3	6
28199627	Hypothetical protein	PD1756	OM	34	35.8	7
28199090	ABC transporter phosphate binding protein	pstS	U	39	26.4	6

28199405	Peptidyl-prolyl cis-trans isomerase	PD1525	OM	25	53.0	10
28199549	Bacterioferritin	bfr	C	18	54.5	6
28199381	3-oxoacyl-(acyl carrier protein) synthase II	fabB	CM	43	46.5	9
28198372	50S ribosomal protein L15	rplO	C	15	53.1	6
28199723	Putative uncharacterized protein	PD1854	U	23	29.9	5
28199057	UDP-glucose dehydrogenase	ugd	C	50	26.9	9
28198353	50S ribosomal protein L3	rplC	C	23	38.1	6
28198346	ATP synthase subunit alpha	atpA	C	56	22.3	7
28199146	Enolase	eno	U	46	30.5	7
77747646	Phosphoenolpyruvate synthase	ppsA	C	87	25.8	13
28198403	Glutathione S-transferase	gst	C	23	48.3	6
28198480	Putative uncharacterized protein	PD0570	OM	44	20.0	7
28198937	Organic hydroperoxide resistance protein	ohr	C	15	67.8	8
28198389	Histone-like protein	hup	C	10	76.6	5
28198923	Glutamine synthetase	glnA	C	52	25.4	7
77747643	GTP-binding elongation factor protein	typA	CM	68	21.4	9
28199916	Transcription termination factor Rho	rho	C	48	18.5	6
28198370	30S ribosomal protein S5	rpsE	C	19	45.8	6
28199872	Transcription antitermination protein nusG	nusG	C	21	58.9	7
28199293	Glutaredoxin-like protein	grx	C	33	28.6	6
28199554	Two-component system, regulatory protein	popP	C	25	27.8	5
28198378	50S ribosomal protein L17	rplQ	C	14	50.0	6
28199609	Putative uncharacterized protein	PD1736	U	18	44.0	5
28198663	Peptidoglycan-associated outer membrane lipoprotein	pcp	OM	16	34.0	4
28198177	NADH dehydrogenase subunit G	nuoG	C	80	16.4	7
28199869	50S ribosomal protein L10	rplJ	C	19	36.6	4
28198354	50S ribosomal protein L4	rplD	C	22	46.5	7
28199172	Periplasmic protease	mucD	P	54	25.1	7
28198021	50S ribosomal protein L19	rplS	C	15	50.7	6
28198032	Carbon storage regulator homolog	csrA	U	8	63.4	3
28199870	50S ribosomal protein L1	rplA	C	24	35.8	4
28198140	Phosphomannose isomerase-GDP-mannose pyrophosphorylase	xanB	C	51	18.0	5
28198657	30S ribosomal protein S9	rpsI	C	14	52.3	5
28199329	Inosine-5'-monophosphate dehydrogenase	guaB	C	52	16.3	4

28199917	Isocitrate dehydrogenase [NADP]	icd	C	81	16.7	6
28198121	Transcription elongation factor nusA	nusA	C	56	12.5	5
28198753	Chemotaxis-related protein kinase	cheA	C	195	4.4	5
28198661	cAMP-regulatory protein	clp	C	26	29.7	5
28198400	50S ribosomal protein L28	rpmB	C	9	48.7	5
28198149	6-phosphofructokinase	pfkA	C	47	26.9	7
28199331	UTP-glucose-1-phosphate uridylyltransferase	gtaB	C	33	19.3	4
28198467	Rod shape-determining protein	mreB	C	39	33.4	8
28199586	TonB-dependent receptor	PD1711	OM	98	14.1	9
28199680	Dihydrolipoamide acetyltranferase	pdhB	C	58	13.8	5
28198358	50S ribosomal protein L22	rplV	C	12	50.5	5
28198688	Multidrug-efflux transporter	acrF	CM	114	8.2	7
28199503	Glyceraldehyde-3-phosphate dehydrogenase	gapA	C	36	30.4	5
28198367	30S ribosomal protein S8	rpsH	C	14	38.6	5
28197993	Signal recognition particle protein	ffh	CM	50	20.4	6
28198752	Pilus biogenesis protein	pilJ	CM	74	16.0	7
28198338	Tryptophan repressor binding protein	wrbA	U	20	22.2	4
28198364	50S ribosomal protein L24	rplX	C	11	44.8	6
77747670	Thiamine biosynthesis protein	thiC	C	66	13.1	5
28199786	Two-component system, regulatory protein	colR	C	25	24.9	3
28198352	30S ribosomal protein S10	rpsJ	C	12	31.1	3
28199201	Chromosome-partitioning protein	parB	C	34	15.2	3
28199877	50S ribosomal protein L25	rplY	C	23	30.0	4
28198018	30S ribosomal protein S16	rpsP	C	10	58.1	5
28199194	5-methyltetrahydropteroyltriglutamate--homocysteine methyltransferase	metE	C	86	9.9	4
28198141	Phosphoglucomutase	xanA	C	49	23.6	6
28199678	Outer membrane protein	ompW	OM	23	32.6	4
161484696	Aconitate hydratase	rpfA	C	101	7.6	4
28199621	Serine hydroxymethyltransferase	glyA	C	45	18.9	6
28199385	Pyruvate dehydrogenase E1 component	aceE	C	100	14.8	9
28198233	Putative uncharacterized protein	PD0312	P	10	50.0	2
28198174	NADH dehydrogenase subunit D	nuoD	C	49	11.0	3
28198112	Preprotein translocase subunit SecD	secD	CM	68	6.6	3
28198125	30S ribosomal protein S15	rpsO	C	10	46.5	4

77747692	GumK protein	gumK	C	31	24.6	4
28198655	50S ribosomal protein L31 type B	rpmE2	U	9	63.8	4
28199865	30S ribosomal protein S12	rpsL	C	14	37.9	3
28197947	DNA polymerase III subunit beta	dnaN	C	42	15.6	4
28198567	Thiol:disulfide interchange protein	dsbA	P	28	24.6	5
28198030	Recombinase A	recA	C	38	18.2	4
28198270	Succinate dehydrogenase iron-sulfur protein	sdhB	CM	30	16.1	2
28198450	Putative uncharacterized protein	PD0540	U	15	43.8	4
28199283	Isopropylmalate isomerase large subunit	leuC	C	51	11.4	3
28199536	Lipoprotein precursor	vacJ	OM	39	13.1	4
28198316	Carbamoyl phosphate synthase large subunit	carB	U	117	7.7	4
28198298	Phage-related protein	PD0381	U	11	55.9	4
28198363	50S ribosomal protein L14	rplN	C	13	34.4	6
28199812	30S ribosomal protein S6	rpsF	C	16	33.6	4
28198387	ATP-dependent protease ATP-binding subunit	clpX	C	47	19.2	5
28198664	Dihydrolipoamide dehydrogenase	lpd	C	51	9.4	3
28199926	TonB-dependent receptor	cirA	OM	96	5.8	3
28198360	50S ribosomal protein L16	rplP	C	15	27.7	3
28199722	Zinc protease	PD1853	P	106	6.8	4
28199094	Phosphate regulon transcriptional regulator	phoU	C	27	10.8	2
28198033	Oligopeptidase A	prlC	C	76	7.3	3
28199633	Methionyl-tRNA formyltransferase	fmt	C	33	15.6	3
28198097	Phosphoribosylaminoimidazole-succinocarboxamide synthase	purC	C	34	17.2	3
28198774	Superoxide dismutase (Mn)	sodA	P	26	25.7	4
28198348	ATP synthase subunit b	atpF	CM	17	31.4	3
28198428	Putative uncharacterized protein	PD0518	U	179	2.4	3
28198416	Putative uncharacterized protein	PD0504	U	14	19.4	3
28198374	30S ribosomal protein S13	rpsM	C	14	34.7	4
28198334	TldD protein	tldD	C	51	9.1	3
28199839	Isocitrate dehydrogenase	icdA	C	36	27.5	6
28198362	30S ribosomal protein S17	rpsQ	C	10	39.3	3
28198195	Malic enzyme	maeB	C	83	5.9	2
28199783	Threonyl-tRNA synthetase	thrS	C	73	11.3	4
28198563	Glutamate-cysteine ligase precursor	gshI	C	51	9.7	4

28199282	Isopropylmalate isomerase small subunit	leuD	C	24	27.4	4
28198573	ATP-dependent Clp protease subunit	clpA	C	84	4.4	2
28199612	Bacterioferritin comigratory protein	bcp	U	18	30.2	4
28198746	Glycyl-tRNA synthetase beta subunit	glyS	C	80	9.3	3
28198052	Arginyl-tRNA synthetase	argS	C	63	10.3	4
28198187	Porin O precursor	oprO	OM	45	13.6	4
28199811	30S ribosomal protein S18	rpsR	C	9	40.8	2
28198173	NADH dehydrogenase subunit C	nuoC	C	28	11.6	2
28198336	PmbA protein	pmbA	C	48	10.3	2
28199089	Cation:proton antiporter	ybaL	CM	60	10.8	4
28199854	Superoxide dismutase	sodM	P	23	22.7	2
28198116	3-methyl-2-oxobutanoate hydroxymethyltransferase	panB	C	29	21.3	4
28198371	50S ribosomal protein L30	rpmD	U	7	30.2	2
28199290	Alcohol dehydrogenase	yahK	C	37	23.8	5
28198252	Ribosome-recycling factor	frr	C	21	33.5	4
28199681	Dihydrolipoamide dehydrogenase	lpdA	C	64	7.5	3
28199371	Elongation factor P	efp	C	21	21.8	2
28199871	50S ribosomal protein L11	rplK	C	15	12.0	1
28198658	50S ribosomal protein L13	rplM	C	16	29.6	3
28198503	RNA polymerase sigma factor	rpoD	C	70	6.6	3
28198369	50S ribosomal protein L18	rplR	C	13	29.4	3
28199794	Pilus biogenesis protein	pilB	C	63	8.7	3
28199040	Twitching motility protein	pilU	C	42	12.0	3
28198527	DNA-binding related protein	dps	C	19	30.9	3
28199504	Adenylosuccinate synthetase	purA	C	47	9.5	3
28199502	Nucleoside diphosphate kinase	ndk	EC	16	22.0	3
28198150	Adenylate kinase	adk	C	20	23.5	2
28198459	Electron transfer flavoprotein ubiquinone oxidoreductase	etfD	U	60	4.6	3
28198375	30S ribosomal protein S11	rpsK	C	14	26.2	2
28198185	3-oxoacyl-[ACP] reductase	fabG	C	26	23.2	3
28198388	ATP-dependent serine proteinase La	lon	C	92	3.9	3
28198936	ATPase	PD1039	C	45	8.5	2
28198848	Fumarate hydratase	fumB	C	55	8.1	2
77747623	Phospho-2-dehydro-3-deoxyheptonate aldolase	aroG	C	41	13.9	3

28198404	Malate dehydrogenase	mdh	U	35	35.4	6
28198517	Aspartate-semialdehyde dehydrogenase	asd	C	37	16.3	2
28197956	Biopolymer transport ExbD1	exbD1	CM	15	25.2	2
77747689	Polysaccharide export protein	mrp	CM	32	6.6	1
28198960	Preprotein translocase subunit SecB	secB	C	19	43.0	4
28197977	Transcription-repair coupling factor	mfd	U	132	4.4	3
28199058	Peptidyl-prolyl cis-trans isomerase	PD1168	OM	25	14.2	3
28198433	Small conductance mechanosensitive ion channel	yggB	CM	34	19.3	3
28198368	50S ribosomal protein L6	rplF	C	19	11.4	2
77747720	4-hydroxy-3-methylbut-2-en-1-yl diphosphate synthase	ispG	C	45	15.8	4
28199782	Translation initiation factor IF-3	infC	C	21	12.8	2
28199704	Peptidyl-prolyl cis-trans isomerase	surA	P	51	10.6	3
28198847	Aspartyl-tRNA synthetase	aspS	C	66	10.4	3
28198532	Cytochrome O ubiquinol oxidase subunit I	cyoB	CM	74	2.9	2
28199323	50S ribosomal protein L27	rpmA	C	9	25.9	2
28199511	DNA-binding protein	bbh3	U	14	33.1	3
28198444	Poly(Hydroxyalcanoate) granule associated protein	phaF	C	20	17.3	2
77747658	NAD-glutamate dehydrogenase	gdhA	U	186	1.6	2
28199878	Ribose-phosphate pyrophosphokinase	prs	C	35	5.9	1
77747631	Phosphomannomutase	algC	C	50	8.8	2
28198330	Outer membrane protein	omp28	P	26	11.6	2
28198345	ATP synthase gamma chain	atpG	C	32	11.5	2
28198558	Phosphoribosylformylglycinamidine synthase	purL	C	144	5.7	4
28198025	Succinyl-diaminopimelate desuccinylase	dapE	C	41	10.9	2
28198031	Alanyl-tRNA synthetase	alaS	C	96	2.4	1
28198600	ABC transporter ATP-binding protein	ynhD	C	31	9.7	2
28198952	Outer membrane protein Slp	slp	OM	18	20.0	2
28199569	Fimbrial assembly membrane protein	pilN	CM	25	14.1	2
28199623	Probable malate:quinone oxidoreductase	mqo	C	63	11.7	4
77747685	Catalase-peroxidase	katG	C	84	6.3	3
28198361	50S ribosomal protein L29	rpmC	U	8	26.2	2
28198638	General secretory pathway protein E	xpsE	C	65	5.3	2
28198068	Tyrosyl-tRNA synthetase	tyrS	C	47	6.5	1

28198733	Bifunctional purine biosynthesis protein purH	purH	C	57	8.0	2
28198325	Diaminopimelate decarboxylase	lysC	C	95	4.3	3
28199113	Inorganic pyrophosphatase	ppa	C	20	16.9	2
28199043	Putative uncharacterized protein	PD1151	OM	31	16.6	3
28199560	Chaperone protein ClpB	clpB	C	96	5.2	3
28198095	Acetyl-coenzyme A carboxylase carboxyl transferase subunit alpha	accA	C	36	19.1	4
28199809	Chromosome segregation protein	smc	C	131	4.3	2
28198800	2,3-bisphosphoglycerate-dependent phosphoglycerate mutase	gpmA	C	29	13.3	2
28198251	Undecaprenyl pyrophosphate synthase	uppS	C	29	9.8	2
28197982	Nitric oxide dioxygenase	hmp	C	44	8.1	2
28199255	Heat shock protein GrpE	grpE	C	19	26.7	2
28198261	6-phosphogluconolactonase	pgl	U	26	26.4	4
28199883	Peptide chain release factor 1	prfA	C	41	11.1	2
28198411	Colicin V secretion ABC transporter ATP-binding protein	cvaB	CM	79	7.5	2
28199563	Putative cytochrome P450 133B1	CYP133 B1	CM	45	21.6	4
28198026	Asparagine synthase B	asnB	C	63	8.3	3
28199369	3-demethylubiquinone-9 3-methyltransferase	ubiG	C	27	11.8	2
28199383	3-oxoacyl-[ACP] reductase	fabG	C	25	17.0	2
28198366	30S ribosomal protein S14	rpsN	C	12	18.8	2
28198213	Argininosuccinate synthase	argG	C	44	11.5	3
28199115	Thioredoxin domain-containing protein	trx	C	38	9.7	2
28198011	Dihydroxy-acid dehydratase	ilvD	C	65	7.2	3
28199318	Isoleucyl-tRNA synthetase	ileS	C	106	5.1	4
28199677	Membrane protein	PD1806	CM	29	13.4	2
28197999	Fimbrial subunit	PD0062	EC	19	34.8	2
28199334	Putative uncharacterized protein	PD1453	C	44	8.9	2
28199202	Chromosome partitioning protein	parA	CM	29	17.0	2
77747669	Quinolinate synthase A	nadA	C	37	16.5	3
77747710	(Dimethylallyl)adenosine tRNA methylthiotransferase miaB	miaB	C	55	8.7	2
28198405	Peptidyl-prolyl cis-trans isomerase	ppiB	C	18	30.5	3
28199780	50S ribosomal protein L20	rplT	C	14	16.8	2
28199573	Ribokinase	rbsK	C	33	9.1	2

28199399	Peptidyl-prolyl cis-trans isomerase	slyD	C	18	23.9	2
28199204	Seryl-tRNA synthetase	serS	C	47	9.6	3
28198633	Proline iminopeptidase	pip	C	36	9.9	3
28199173	GTP-binding protein LepA	lepA	CM	67	8.3	3
28198562	Acetylornithine aminotransferase	argD	C	44	7.3	2
28199705	LPS-assembly protein lptD	lptD	OM	91	2.9	2
28198958	Putative uncharacterized protein	PD1063	OM	21	32.1	3
28198323	Regulator of pathogenicity factors	rpfB	CM	63	7.0	3
28198668	Adenylosuccinate lyase	purB	C	51	7.0	2
28199552	S-adenosylmethionine synthase	metK	C	44	8.4	2
77747688	Putative uncharacterized protein	PD1329	U	15	16.0	2
28199860	Putative uncharacterized protein	PD1994	CM	78	6.1	3
28198652	Subunit F of alkyl hydroperoxide reductase	ahpF	CM	57	8.3	3
28199377	Type IV fimbriae assembly protein	pilZ	U	13	32.2	2
28199730	Cell division protein	ftsZ	C	42	12.9	2
28199351	Putative uncharacterized protein	PD1470	C	28	10.9	2
28198974	DNA mismatch repair protein mutS	mutS	C	97	4.4	2
77747682	Ferric enterobactin receptor	bfeA	OM	87	6.8	2
28199645	Stringent starvation protein A	sspA	C	24	10.9	2
28198552	UDP-N-acetylglucosamine 1-carboxyvinyltransferase	murA	C	45	11.3	2
28199572	Voltage-gated potassium channel beta subunit	tas	C	36	10.9	2
28198569	High frequency lysogenization protein hflD homolog	hflD	U	22	19.1	2
28198623	Sulfate adenylyltransferase subunit 2	cysD	C	35	7.6	2
28199157	Threonine synthase	thrC	C	46	4.9	2
28198591	Glucosamine--fructose-6-phosphate aminotransferase	glmS	C	36	8.4	2
28197957	Biopolymer transport ExbD2 protein	exbD2	CM	15	32.4	2
28199453	Recombination-associated protein rdgC	rdgC	C	34	9.9	2
28199717	Glutamyl-tRNA synthetase	gltX	C	53	5.4	2
28199174	Signal peptidase I	lepB	CM	30	8.6	2
28199773	Virulence regulator	xrvA	U	15	33.6	2
28198707	Putative uncharacterized protein	PD0802	U	20	15.7	2
28198260	6-phosphogluconate dehydratase	edd	C	67	4.8	2
28199814	Asparaginyl-tRNA synthetase	asnS	C	53	5.8	2

28199427	Cystathionine beta-synthase	cysM	C	50	11.0	2
28198357	30S ribosomal protein S19	rpsS	C	10	25.8	2
28199280	GumB protein	gumB	U	24	11.1	2
28198667	Putative uncharacterized protein	PD0761	C	48	7.8	2
28198559	Disulfide isomerase	dsbC	P	28	14.9	3
28198605	Putative uncharacterized protein	PD0697	CM	11	18.6	2
28198194	C4-dicarboxylate transport protein	dctA	CM	48	5.3	2
28199052	Putative uncharacterized protein	PD1162	C	47	3.0	2
28199241	P-protein	pheA	C	42	6.4	2
28198038	Valyl-tRNA synthetase	valS	C	111	3.2	2
28198855	Putative uncharacterized protein	PD0956	U	37	8.4	2
28199932	Type I restriction-modification system DNA methylase	hsdM	C	89	4.0	2
28198322	Response regulator	rpfG	CM	44	8.1	2

1: Protein accesion number at NCBI database (http://www.ncbi.nlm.nih.gov/);
2: Protein localization as prediceted by PSORTb v. 3.0.2 Subcellular Localization Prediction Tool (http://www.psort.org/psortb/; C: Cytoplasmic; CM: Cytoplasmic Membrane; OM: Outer Membrane; P: Periplasmic; EC: Extracellular; U: Unknown);
3: Theoretical protein molecular weight shown in kDa.

References

1. Combet, C., Jambon, M., Deleage, G. & Geourjon, C. Geno3D: automatic comparative molecular modelling of protein. *Bioinformatics* **18**, 213-214 (2002).
2. Konagurthu, A.S., Whisstock, J.C., Stuckey, P.J. & Lesk, A.M. MUSTANG: a multiple structural alignment algorithm. *Proteins* **64**, 559-574 (2006).
3. Larkin, M.A. et al. Clustal W and Clustal X version 2.0. *Bioinformatics* **23**, 2947-2948 (2007).
4. Aparna, G., Chatterjee, A., Sonti, R.V. & Sankaranarayanan, R. A cell wall-degrading esterase of Xanthomonas oryzae requires a unique substrate recognition module for pathogenesis on rice. *Plant Cell* **21**, 1860-1873 (2009).
5. Gouy, M., Guindon, S. & Gascuel, O. SeaView version 4: A multiplatform graphical user interface for sequence alignment and phylogenetic tree building. *Molecular biology and evolution* **27**, 221-224 (2010).
